# Future Use of AI in Diagnostic Medicine: 2-Wave Cross-Sectional Survey Study

**DOI:** 10.2196/53892

**Published:** 2025-02-27

**Authors:** Bernardo Pereira Cabral, Luiza Amara Maciel Braga, Carlos Gilbert Conte Filho, Bruno Penteado, Sandro Luis Freire de Castro Silva, Leonardo Castro, Marcelo Fornazin, Fabio Mota

**Affiliations:** 1 Cellular Communication Laboratory Oswaldo Cruz Institute Oswaldo Cruz Foundation Rio de Janeiro Brazil; 2 Department of Economics Faculty of Economics Federal University of Bahia Salvador Brazil; 3 Department of Economics Federal University of Santa Maria Santa Maria Brazil; 4 Fiocruz Strategy for the 2030 Agenda Oswaldo Cruz Foundation Rio de Janeiro Brazil; 5 National Cancer Institute Rio de Janeiro Brazil; 6 Graduate Program in Management and Strategy Federal Rural University of Rio de Janeiro Seropedica Brazil; 7 National School of Public Health Oswaldo Cruz Foundation Rio de Janeiro Brazil

**Keywords:** artificial intelligence, AI, diagnostic medicine, survey research, researcher opinion, future

## Abstract

**Background:**

The rapid evolution of artificial intelligence (AI) presents transformative potential for diagnostic medicine, offering opportunities to enhance diagnostic accuracy, reduce costs, and improve patient outcomes.

**Objective:**

This study aimed to assess the expected future impact of AI on diagnostic medicine by comparing global researchers’ expectations using 2 cross-sectional surveys.

**Methods:**

The surveys were conducted in September 2020 and February 2023. Each survey captured a 10-year projection horizon, gathering insights from >3700 researchers with expertise in AI and diagnostic medicine from all over the world. The survey sought to understand the perceived benefits, integration challenges, and evolving attitudes toward AI use in diagnostic settings.

**Results:**

Results indicated a strong expectation among researchers that AI will substantially influence diagnostic medicine within the next decade. Key anticipated benefits include enhanced diagnostic reliability, reduced screening costs, improved patient care, and decreased physician workload, addressing the growing demand for diagnostic services outpacing the supply of medical professionals. Specifically, x-ray diagnosis, heart rhythm interpretation, and skin malignancy detection were identified as the diagnostic tools most likely to be integrated with AI technologies due to their maturity and existing AI applications. The surveys highlighted the growing optimism regarding AI’s ability to transform traditional diagnostic pathways and enhance clinical decision-making processes. Furthermore, the study identified barriers to the integration of AI in diagnostic medicine. The primary challenges cited were the difficulties of embedding AI within existing clinical workflows, ethical and regulatory concerns, and data privacy issues. Respondents emphasized uncertainties around legal responsibility and accountability for AI-supported clinical decisions, data protection challenges, and the need for robust regulatory frameworks to ensure safe AI deployment. Ethical concerns, particularly those related to algorithmic transparency and bias, were noted as increasingly critical, reflecting a heightened awareness of the potential risks associated with AI adoption in clinical settings. Differences between the 2 survey waves indicated a growing focus on ethical and regulatory issues, suggesting an evolving recognition of these challenges over time.

**Conclusions:**

Despite these barriers, there was notable consistency in researchers’ expectations across the 2 survey periods, indicating a stable and sustained outlook on AI’s transformative potential in diagnostic medicine. The findings show the need for interdisciplinary collaboration among clinicians, AI developers, and regulators to address ethical and practical challenges while maximizing AI’s benefits. This study offers insights into the projected trajectory of AI in diagnostic medicine, guiding stakeholders, including health care providers, policy makers, and technology developers, on navigating the opportunities and challenges of AI integration.

## Introduction

### Background

Artificial intelligence (AI) is a broad term that covers various technologies such as machine learning, natural language processing, neural networks, and specialized rule-based systems [1,2]. It has emerged as a new paradigm in health care [3-7], with applications in disease risk assessment, treatment outcomes prediction, medical complication prevention or mitigation, clinical research, drug development, and patient care [8]. The AI market in health care is expanding rapidly, with a market size of US $26.69 billion in 2024 and projected to grow to approximately US $613.81 billion by 2034 [9]. In the United States alone, the Food and Drug Administration received <500 submissions between 2016 and 2022 [10].

Specifically, AI is expected to promote major changes in diagnostic medicine [4,11-13]. This branch of medicine focuses on identifying diseases and medical conditions by evaluating symptoms, physical signs, and test results [14,15]. Expected benefits of adopting AI include more reliable diagnoses, reduced screening costs, improved health care access, and reduced physician workload [13,16-18]. AI’s ability to analyze large datasets quickly and accurately can also facilitate early detection of diseases [19-21] and support personalized treatment decisions [22], offering opportunities to improve patient outcomes significantly [23]. In addition, AI-driven tools can assist in managing diagnostic workflows by automating routine tasks [23,24], allowing physicians to focus on more complex cases. These benefits are especially desirable as the demand for imaging diagnoses has been rising faster than the supply of physicians for decades [3,11,17].

However, several factors can influence the direction and speed of changes in diagnostic medicine. Among these, one such factor is ethical and regulatory issues, which include difficulties in accessing and sharing databases with patient information [1,19], data validation and auditing [20-22], uncertainties about legal liability for the use of algorithms [23-25], and bias of algorithmic underrepresentation [20,26]. Technical challenges, such as the integration of AI systems into existing health care infrastructure [27] and the need for ongoing training of AI models to adapt to new medical knowledge [28], also pose significant hurdles. In addition, the lack of standardization in AI development and deployment can lead to inconsistent performance across different clinical settings [29,30]. All these factors mean that the future of AI use in diagnostic medicine is still quite uncertain, despite its potential.

### Objectives

What, then, is the future of AI use in diagnostic medicine? This study aimed to anticipate future possibilities of AI use in diagnostic medicine, comparing researchers’ expectations over time. For this purpose, we conducted 2 global cross-sectional surveys with authors of recent scientific publications on AI and diagnostic medicine, indexed in the Web of Science (WoS) Core Collection database. Some previous studies provided literature reviews to anticipate expected changes in health care using AI [1,3,8,12,18,27,28,31-36]. More specific studies on AI applications in diagnostic medicine focused on specific diagnostic tools, such as medical imaging [29], electrocardiograms [30], or cancer classification [37]. However, none of them comprehensively addressed aspects related to the future use of AI in diagnostic medicine and attempted to follow up on possible changes in the expected future. Our study addresses this gap by surveying the opinions of >3000 researchers worldwide at 2 different moments, in September 2020 and February 2023, with 28 months in between. A similar approach has been adopted in other studies to assess changes in attitudes or perceptions over time [38-41]. The survey participants shared their expectations on aspects related to (1) the probability of occurrence of events pointed out by the scientific literature, (2) the integration between AI and various types of diagnostic instruments, and (3) the main barriers to the use of AI in diagnostic medicine.

This study provides a unique perspective on the expected changes in diagnostic medicine due to the integration of AI and diagnostic medicine. Our findings may help health care providers and policy makers make informed decisions on integrating AI into clinical practice. In addition, our study may interest those investing in research and development and those expected to apply AI technologies in diagnostic medicine.

## Methods

### Literature Review

A literature review was conducted to explore the challenges and opportunities of AI applications in diagnostic medicine and build the questionnaire. We searched for recent articles, editorials, and reviews on this topic in WoS-indexed journals. The publications were identified using the search strategy provided in Textbox 1.

**The search strategy used to identify publications in Web of Science (WoS)–indexed journals.**

TS=(“Artificial intelligence” OR “Computational Intelligence” OR “Machine Intelligence” OR “Computer Reasoning” OR AI OR “Computer Vision System*”) AND TS=(future* OR foresight* OR forthcoming* OR prospective* OR imminent*) AND TS=(diagnostics OR medicine OR “clinical practice”)

AND LANGUAGE: (English) AND DOCUMENT TYPES: (Article OR Editorial Material OR Review)

Indexes: SCI-Expanded; timespan: 2015 to 2020

The search strategy combined thesaurus terms related to AI, medical diagnosis obtained from the Medical Subject Headings (MeSH) of the US National Library of Medicine, as well as free-text words for terms related to the future. In the WoS advanced search mode, we used the tag topic to search for those terms in the titles, abstracts, and keywords of articles, editorial materials, and reviews published between 2015 and May 2020 and indexed in the Science Citation Index (SCI)-Expanded. The SCI-Expanded was used to retrieve documents published in journals of sciences, and the period was set to obtain recent information on applications of AI in diagnostic medicine.

The search was conducted in May 2020 and recovered 536 publication records, imported in plain text format to the data- and text-mining software VantagePoint (version 11.0; Search Technology Inc). After reading their titles and abstracts, we selected 65 publication records for further analysis. These records were then imported into the software Citavi (version 6.1; Swiss Academic Software GmbH), where we read the texts fully and managed the references. Of these 65 publications, we selected 27 (41%) that formed the basis of the literature review and the survey questionnaire [1-3,5,8,11,16-18,20,23,29, 33-35,42-52]. As the questionnaire’s content could not be changed to allow for comparing the results from both waves, we did not update the literature review.

### Survey Design

We followed the Checklist for Reporting Results of Internet E-Surveys [53], and the detailed checklist is provided in Multimedia Appendix 1. The questionnaire considered a horizon of 10 years and was divided into 6 parts. The first part introduced the study’s aim, data collection and treatment procedures, anonymity and confidentiality guarantees, voluntary participation conditions, and informed consent. The second part asked about the respondents’ knowledge regarding diagnostic medicine. Only those who reported having good or some knowledge qualified for the survey. Those who reported no knowledge were disqualified and excluded from the survey; hence, they were prevented from proceeding further with the main questionnaire. The third part asked about the respondents’ expectations of AI’s impact on diagnostic medicine in terms of (1) radical changes, (2) future outcomes (eg, reducing physicians’ workload), and (3) integration with diagnostic instruments (eg, interpreting brain magnetic resonance imaging). The fourth part asked about the general barriers to using AI in diagnostic medicine. The respondents had to choose 1 of the 4 options as the most important barrier (eg, the difficulty of incorporation into clinical practice). On the basis of their choice, they were directed to a question about specific barriers within that category, where they had to select one as the most important barrier (eg, conflicts between AI and other clinical strategies). The bibliographic references for each question in these parts of the questionnaire are listed in Multimedia Appendix 2.

The fifth and sixth parts were optional and complementary to the questionnaire as they were not part of the survey’s core. Thus, the data collected were not considered when calculating the number of fully completed questionnaires. The fifth part was an open-ended question where respondents could provide new information about the use of AI in diagnostic medicine or submit comments on the survey. The last part consisted of 5 demographic questions. As the results of this type of study were not influenced by the respondents’ demographics [54-57], these questions were included to provide an overview of respondents’ academic degrees, professional occupations, institutional affiliation, professional experience, and the region where they live. The questionnaire consisted of 10 pages.

### Respondent Recruitment

Respondent recruitment followed a detailed and systematic process. The first wave (W1) and second wave (W2) respondents were authors of articles or review articles related to AI and diagnostic medicine published in peer-reviewed journals indexed in WoS’s SCI-Expanded between January 1, 2015, and September 20, 2020 (W1), and between September 21, 2020, and December 30, 2022 (W2). To identify the participants, we used the query provided in Textbox 2 using the WoS advanced search mode.

**The query used to identify participants using Web of Science advanced search mode.**

TS=(“Artificial intelligence” OR “Computational Intelligence” OR “Machine Intelligence” OR “Computer Reasoning” OR AI OR “Computer Vision System*”) AND TS=(Diagnos* OR Medicine OR Medical OR Ultrasound* OR Ultrasonograph* OR “Ultrasonic Diagnos*” OR Echotomograph* OR Echograph* OR “Radionuclide Imaging” OR “Radioisotope Scanning” OR radiograph* OR Roentgenograph* OR “X-ray”)

Refined by DOCUMENT TYPES: (ARTICLE OR REVIEW)

Indexes: SCI-Expanded

The query was designed to capture a broad range of AI applications in diagnostic medicine. The thesaurus terms related to AI and diagnostic medicine were collected in MeSH. The query was set to retrieve recently published peer-reviewed articles and review articles. In W1, conducted in September 2020, we retrieved 15,084 publication records. These records were processed using the VantagePoint software to extract 23,053 authors’ emails. The extracted emails were compiled into a CSV file containing the authors’ names, emails, and titles of their respective articles. An in-house Python (Python Software Foundation) script was used to cross-check and link 80.11% (18,469/23,053) of the extracted emails to their respective authors, enabling a more personalized approach to sending the survey invitations.

Similarly, in W2, conducted in December 2022, we retrieved 35,146 publication records, leading to 65,149 author emails. We linked 80.03% (52,138/65,149) of the emails to their respective authors using the same process. This consistent approach ensured the recruitment of a wide-reaching and diverse sample of respondents. The use of personalized invitations, sent directly to authors, aimed to enhance the engagement and response rate. The recruitment process was systematic and designed to ensure the survey’s reach across key stakeholders in the AI and diagnostic medicine fields. The procedures used were similar to those of other studies using the same method [39,54,56-60], especially those with 2 survey waves [38]. A summarized and visual representation of this process of recruiting respondents is provided in Multimedia Appendix 3.

In choosing this methodological approach, we aimed to capture the rapidly evolving landscape of AI in diagnostic medicine. The decision to conduct 2 waves of surveys with different cohorts was driven by the need to reflect the dynamic nature of this field. AI technologies and their applications in medicine are advancing at a fast pace, and by expanding the cohort in W2, we sought to provide a broader and more current perspective on these developments. This approach allowed us to present a comprehensive understanding of the trends, challenges, and expectations within the AI and diagnostic medicine community over time.

The inclusion of respondents from engineering and computer science backgrounds was intentional, reflecting the interdisciplinary nature of AI development and its application in diagnostic medicine. The integration of AI into clinical practice is not solely a clinical challenge but also a technological one [34]. Engineers and computer scientists play a crucial role in advancing the algorithms and systems that underpin AI applications in diagnostics. Their insights into technological feasibility, innovation potential, and future directions are invaluable for understanding how these tools might evolve and impact clinical practice. Thus, their contributions are essential to forming a well-rounded perspective on the future of AI use in diagnostic medicine.

### Data Collection Procedures

The list of respondents was imported into the web-based survey platform SurveyMonkey (SurveyMonkey Inc), where the questionnaire was designed and the survey was conducted. After uploading the list of respondents, the number of emails was reduced due to bounced emails and opted-out respondents (people who previously chose not to participate in surveys conducted via SurveyMonkey). In W1, the final number was 20,952, whereas in W2, it was 56,480. The personalized email invitations included a direct link to the questionnaire, inviting participants to take part in the survey. The email outlined the purpose of the study, its importance, and instructions for accessing and completing the survey. To encourage participation and improve response rates, reminder emails were sent to nonrespondents every 2 days for a period of 1 week, totaling a maximum of 3 reminders in addition to the invitation to nonrespondents.

In W1, before the formal study, the questionnaire was validated through a pilot study with a random sample of 2000 researchers (2000/20,952, 9.55% of the total emails). In this phase, we evaluated the questionnaire (ie, application routine, consistency, internal logic, completion rate, and response time) and collected feedback from respondents. Of 2000 researchers, 91 (4.55%) who participated in the pilot study did not suggest any changes in the questionnaire. Thus, neither the questionnaire nor the application routine was modified, and the data collected were added to the study results. Following this validation, the full survey was launched to the remaining sample (18,952/20,000, 94.76%), using the same recruitment process for both waves. In W1, the pilot and the formal study were conducted in September 2020. The formal study of W2 was conducted between January and February 2023. To maintain data integrity, each email address was restricted to submitting only 1 response. The SurveyMonkey platform automatically prevented duplicate entries by tracking responses based on individual email addresses. No randomization of questionnaire items was used.

### Ethical Considerations

The questionnaire, invitation, and reminder emails informed the respondents about the survey. Before answering the questionnaire, they were informed that the survey was being conducted for research purposes only, personal or sensitive data would not be collected, answers would not be identified in the results, participation would be voluntary, and informed consent would be provided by answering the questionnaire. Thus, all respondents who participated in this survey gave us their informed consent to use the data collected. Given the voluntary participation, anonymity in the results, and absence of sensitive or personal questions, an examination by an ethics committee was not necessary. This study followed the guidelines set forth by Brazilian Resolution 510 of April 7, 2016 (Official Federal Gazette [61]), which provides an exemption from ethics committee registration and evaluation for public opinion research with unidentified participants. In addition, the study data were anonymous, and there was no compensation for the respondents. Again, these procedures followed previous studies that used the same method [39,54,56-60].

### Statistical Analysis of the Sample

We used the nonparametric marginal homogeneity test at a 5% significance level to compare the responses obtained in both waves and assess any changes in respondents’ expectations over time. This test is typically used to compare nominal data from 2 related samples and determine whether there is a significant difference between their proportions, particularly when the data are not normally distributed [62,63]. It is commonly applied in before-and-after studies [64,65].

In addition to analyzing the differences between W1 and W2 responses, we evaluated the differences between the responses of the 2 knowledge groups (ie, good and some knowledge) for each wave. We used the Mann-Whitney *U* test to identify whether the level of knowledge interfered with the results. The Mann-Whitney *U* test is a nonparametric statistical test applied when data are not normally distributed [63]. It is commonly used to compare 2 independent groups and determine whether there is a statistically significant difference between them [66]. The test is often applied in cross-sectional research studies [67-69].

Out of the 25 questions presented to the respondents, only 1 (4%) revealed a statistical difference between W1 and W2 by the marginal homogeneity test results. In 2022, the respondents anticipated that AI would be used to interpret heart rhythm and provide improved outcomes in a shorter period than expected in 2020. The results of the tests are reported in Multimedia Appendix 4.

The results of the Mann-Whitney *U* test indicated that there was no statistical difference between most of the responses from the good and some knowledge groups. Only 30% (9/30) of the W1 questions and 10% (3/30) of the W2 questions displayed significant variations between the 2 groups. Hence, we opted to present the aggregated results. The responses categorized by knowledge level are provided in Multimedia Appendix 4.

## Results

Table 1 presents an overview of the survey details, including response rates, completed questionnaires, confidence level, margin of error, and the demographics of respondents in W1 and W2. The respondents had a high level of education, with >80% (1039/1245) having a doctoral degree, and the most common occupation was professor or researcher (W1: 901/1039, 72.1% and W2: 1395/2097, 66.5%). Most respondents worked at universities or research organizations (W1: 1007/1251, 80.3% and W2: 1607/2095, 76.7%) and had a varied range of experience, with approximately 30% (383/1249) having 10 to 20 years and 25% (345/1249) to almost 30% (664/2097) having >20 years of experience in both waves. The most common regions of residence were Europe (W1: 458/1251, 36.6% and W2: 849/2099, 40.5%), Asia (W1: 367/1251, 29.3% and W2: 640/2099, 30.5%), and North America (W1: 258/1251, 20.6% and W2: 350/2099, 16.7%).

In addition to the demographic questions, we mapped the publication profile of both participants and nonparticipants of the survey through the metadata of their article records collected in WoS. The results are provided in Multimedia Appendix 5, and it shows that the survey participants and nonparticipants are similar concerning the research areas whose publications have been indexed.

We first asked the respondents about their general expectations of AI’s potential to change diagnostic medicine radically in the future. Most respondents (W1: 965/1410, 68.5% and W2: 1555/2324, 66.9%) anticipated that this would happen within 10 years, while a smaller proportion (W1: 420/1410, 29.7% and W2: 706/2324, 30.1%) expected this to take longer. A minor percentage (W1: 25/1410, 1.8% and W2: 63/2324, 2.7%) believed that such a radical change was unlikely.

**Figure 1:**
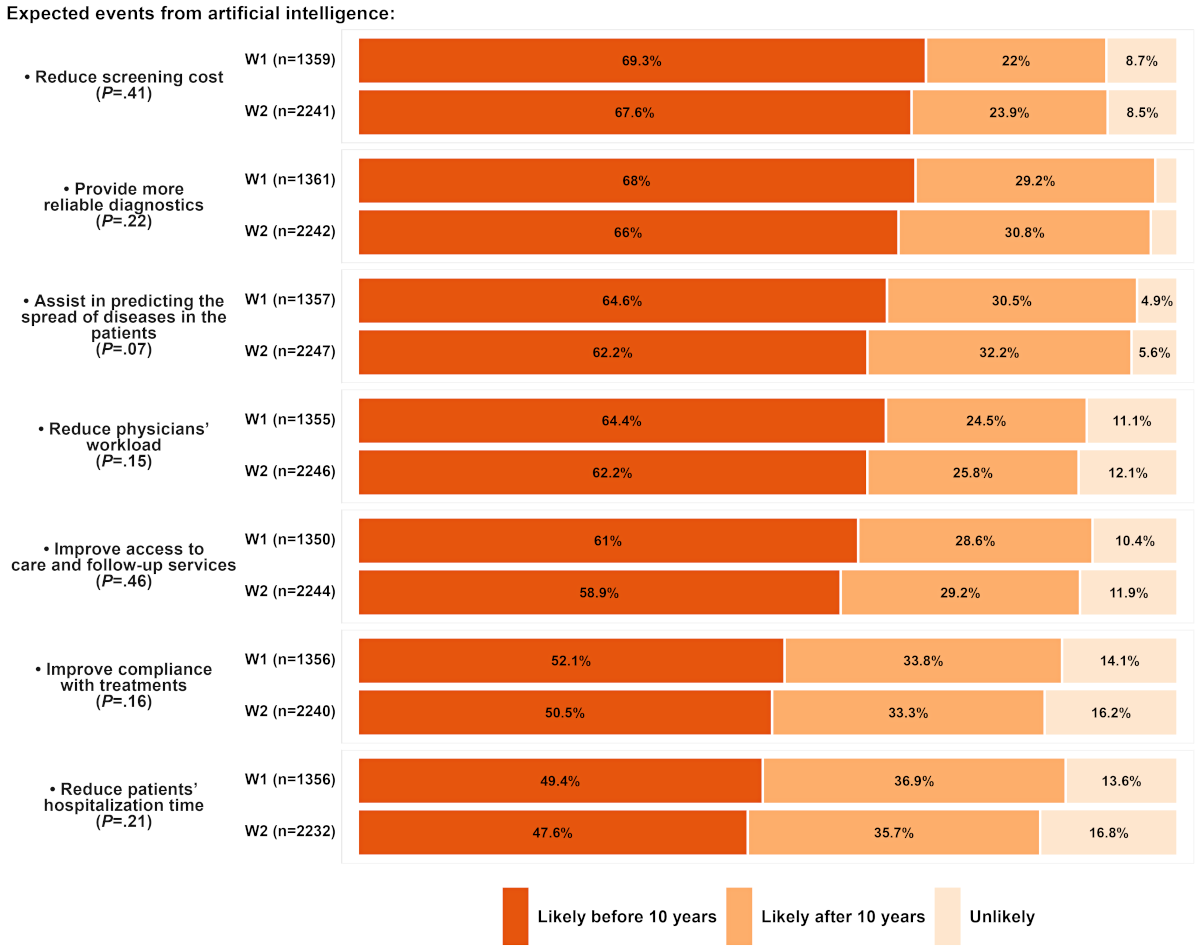
The likelihood of expected future events from artificial intelligence use in diagnostic medicine based on 2 global surveys: wave 1 (WI) in September 2020 and wave 2 (W2) in February 2023. Responses showed the expected impact on screening costs, diagnostic reliability, disease prediction, physician workload, access to care, treatment compliance, and hospitalization time, categorized as likely before 10 years, after 10 years, or unlikely. *P* value refers to the marginal homogeneity test.

The respondents’ expectations of 7 future events resulting from using AI in diagnostic medicine are depicted in Figure 1. Reduction of patients’ hospitalization time (<50% in both waves) and improvement in treatment compliance (>50% in both waves) received the lowest percentages of likely before 10 years in both waves. Most respondents expected a lower screening cost (W1: 942/1360, 69.3% and W2: 1514/2243, 67.6%) and a higher diagnosis reliability (W1: 926/1362, 68% and W2: 1479/2244, 66%) within 10 years. The latter was also the most agreed-upon event, with only approximately 3% (37/1362) of respondents considering its occurrence unlikely. Improvements in treatment compliance and reduction in patients’ hospitalization received the highest percentages of unlikely in both waves.

**Table 1:** Overview of respondent demographics, knowledge level, and survey details for 2 waves of a global cross-sectional survey on artificial intelligence use in diagnostic medicine. Data include number of invitations, response rates, knowledge levels, educational degrees, occupations, institutional affiliations, years of experience, and regional distribution of respondents in wave 1 (WI; September 2020) and wave 2 (W2; February 2023).

				W1, n (%)		W2, n (%)
**Survey summary** **(W1: n=20,952; W2: n=56,480)^a^**						
	Response rate			1622 (7.7)		2606 (4.6)
	Fully completed questionnaires			1208^b^ (5.7)		1724^c^ (3)
**Knowledge level (W1: n=1622; W2: n=2606)**						
	Good knowledge			724 (44.6)		1192 (45.7)
	Some knowledge			706 (43.5)		1160 (44.5)
	No knowledge^d^			192 (11.8)		254 (9.7)
**Demographic information**						
	**Degree of education**					
		Doctoral degree	1039 (83.5)		1717 (82.3)	
		Master’s degree	162 (13)		292 (14)	
		Bachelor’s degree	19 (1.5)		48 (2.3)	
		Associate’s degree	25 (2)		30 (1.4)	
	**Occupation**					
		Professor or researcher	901 (72.1)		1395 (66.5)	
		Physician or clinician	133 (10.6)		276 (13.2)	
		Master’s degree student or PhD^e^ student	137 (11)		238 (11.3)	
		Other	40 (3.2)		62 (3)	
		Public health or health care professional	14 (1.1)		61 (2.9)	
		Manager or executive	23 (1.8)		60 (2.9)	
		Policy maker	1 (0.1)		5 (0.2)	
	**Work institution**					
		University or research organization	1007 (80.5)		1607 (76.7)	
		Hospital or similar organizations	148 (11.8)		315 (15)	
		Industry	69 (5.5)		120 (5.7)	
		Government	27 (2.2)		53 (2.5)	
	**Experience (years)**					
		10 to 20	383 (30.7)		676 (32.2)	
		>20	345 (27.6)		664 (31.7)	
		5 to 10	343 (27.5)		510 (24.3)	
		<5	178 (14.3)		247 (11.8)	
	**Region**					
		Europe	458 (36.6)		849 (40.4)	
		Asia (including the Middle East)	367 (29.3)		640 (30.5)	
		North America (including Central America and the Caribbean)	258 (20.6)		350 (16.7)	
		South America	102 (8.2)		173 (8.2)	
		Africa	34 (2.7)		50 (2.4)	
		Australasia or Pacific Islands	32 (2.6)		37 (1.8)	

^a^Number of participants to whom the survey invitation was sent.

^b^Confidence level of 95% and a margin of error of 2.7%.

^c^Confidence level of 95% and a margin of error of 2.3%.

^d^Respondents with no knowledge were disqualified and prevented from proceeding to the main questionnaire.

^e^PhD: Doctor of Philosophy.

**Figure 2:**
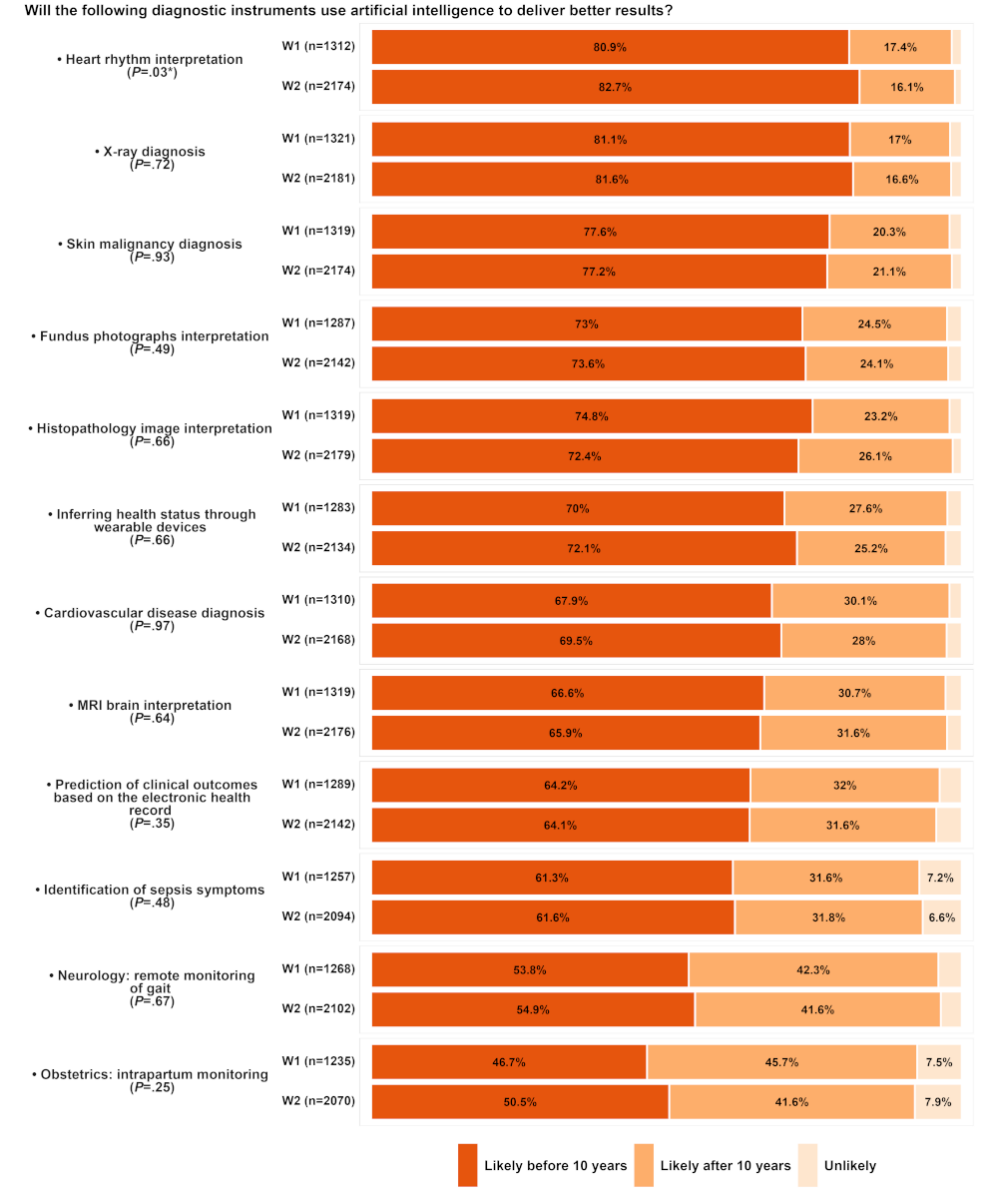
The likelihood of integrating artificial intelligence with various diagnostic instruments to deliver better results, based on global survey responses from wave 1 (W1; September 2020) and wave 2 (W2; February 2023). Instruments included heart rhythm interpretation, x-ray diagnosis, skin malignancy diagnosis, and others. *Statistical significance was at the 5% level. MRI: magnetic resonance imaging.

The respondents’ expectations of integrating AI and 12 selected diagnostic instruments to enhance diagnostic outcomes are illustrated in Figure 2. The 3 most expected integrations were x-ray diagnosis (W1: 1071/1322, 81% and W2: 1787/2183, 81.6%), heart rhythm interpretation (W1: 1062/1313, 80.9% and W2: 1578/2181, 72.4%), and skin malignancy diagnosis (W1: 1024/1320, 77.6% and W2: 1680/2176, 77.2%). Intrapartum monitoring (W1: 577/1235, 46.7% and W2: 1045/2070, 50.5%) and identification of sepsis symptoms (W1: 770/1257, 61.3% and W2: 1290/2095, 61.6%) were the events with the highest percentages of unlikely.

**Figure 3:**
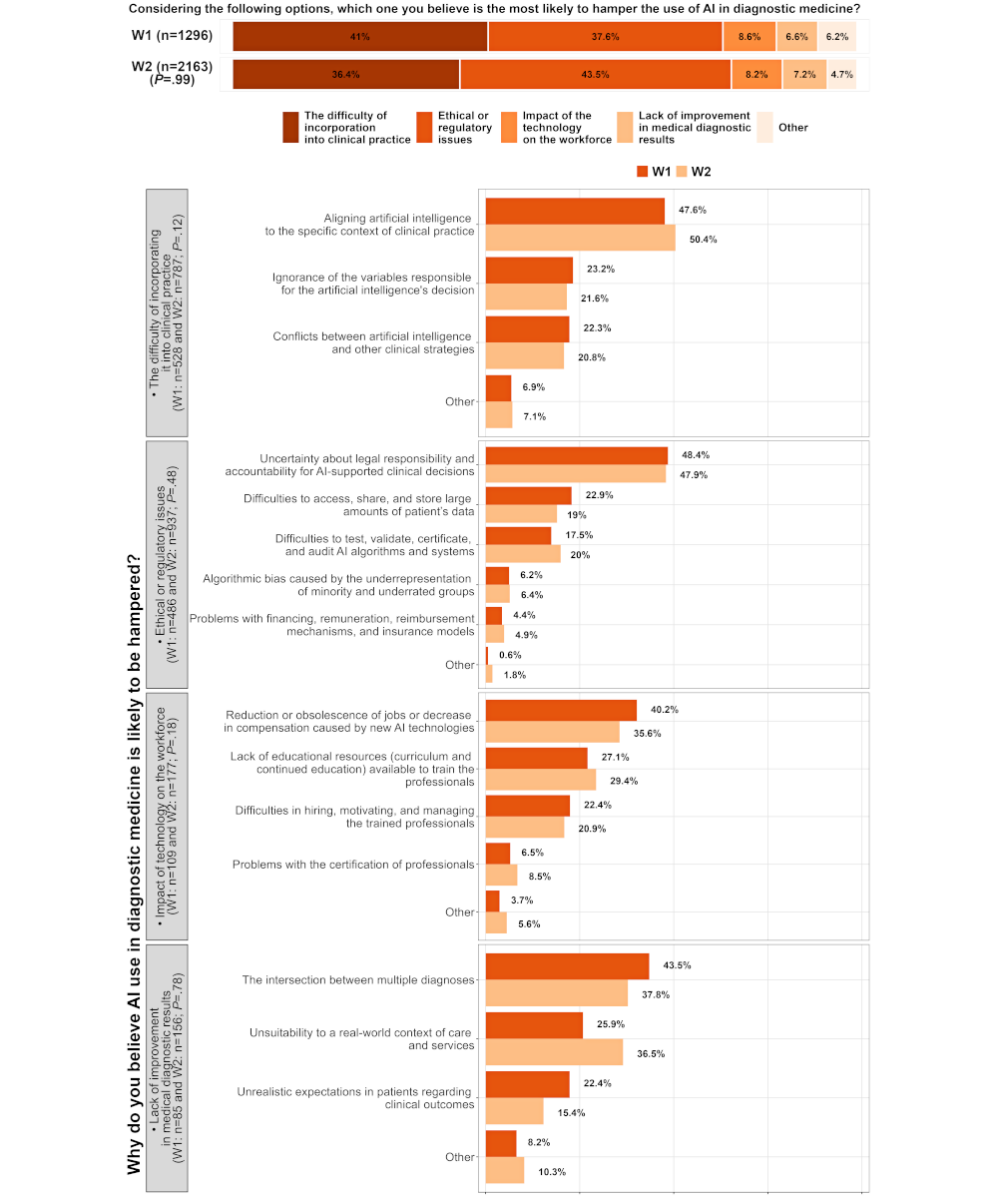
Barriers to artificial intelligence (AI) adoption in diagnostic medicine, comparing wave 1 (WI; September 2020) and wave 2 (W2; February 2023) survey results. Responses addressed clinical, ethical, regulatory, and workforce-related challenges. *P* value refers to the marginal homogeneity test.

The respondents’ perceptions of the main barriers to using AI in diagnostic medicine are shown in Figure 3. The most common barriers in both waves were the difficulty of incorporating AI into clinical practice and ethical or regulatory issues. In W1, these barriers were selected by 41% (531/1296) and 37.6% (487/1296) of the respondents who answered this question, respectively. In W2, ethical or regulatory issues were slightly more prevalent (941/2164, 43.5%) than the difficulty of incorporating AI into clinical practice (788/2164, 36.4%). The impact of AI on the workforce and the lack of improvement in medical diagnostics were less frequently chosen as barriers, with a combined percentage of approximately 15% (W1: 167/1296 and W2: 333/2164) in both waves. The respondents who identified the difficulty of incorporating AI into clinical practice as a barrier also reported its specific related challenges. The most common challenge in both waves was aligning AI to the specific context of clinical practice (W1: 255/535, 47.6% and W2: 399/789, 50.4%).

Similarly, the respondents who identified ethical or regulatory issues as barriers reported their related challenges. The most common challenge in both waves was the uncertainty about legal responsibility and accountability for AI-supported clinical decisions (W1: 235/489, 48.4% and W2: 449/940, 47.8%). When comparing other specific challenges, the one where waves differed the most was the unsuitability of AI to a real-world context of care and services (W1: 22/87, 25.3% and W2: 57/158, 36.1%).

## Discussion

### Principal Findings

Although the span of 28 months may seem a short period to assess changes in researchers’ expectations on a given topic, the case of AI in diagnostic medicine is peculiar. This is an area where transformations are quick, and innovations are the norm [51]. One indicator of this rapid transformation is the surge in publications on this topic. For instance, a quick search on WoS between January 1, 2015, and September 20, 2020, showed >6000 articles on AI in diagnostic medicine. However, between September 21, 2020, and December 30, 2022, this number rose to >12,000, an increase of 113.5% (the search strategies used to identify these publications are the same as those described in Textbox 2).

AI is expected to improve the quality of health care for patients, mainly by addressing patient safety and reducing medical errors [18,70,71]. A study compared deep learning algorithms and physicians in diagnosing skin conditions from photos and dermoscopic images to evaluate how AI can support or automate health care decision-making. The results showed that the algorithms performed better than the average physician [18]. Image reconstruction using deep learning algorithms is another example of how AI can benefit patients. This technique can potentially reduce radiation exposure and the time needed for image acquisition and segmentation in preparation for radiotherapy, which may reduce side effects and material costs [72].

Survey respondents in both waves agreed that AI could positively impact quality over a 10-year time horizon by (1) aiding the prediction of disease progression, (2) increasing diagnostic reliability, and (3) improving treatment compliance. Furthermore, they believed that AI might improve quality in diagnostic medicine in <10 years by producing more reliable, compliable, and predictable outcomes. The answers to the questionnaire (Figure 2) show that AI-based quality improvements are associated with patient outcomes, but the technology may also benefit health care practitioners by reducing human error and fatigue [18,73-75].

Information accuracy is fundamental for achieving better-quality health care outcomes. However, technology and information accuracy may become a barrier to adopting AI [20,76]. For example, most of the researchers (W1: 255/535, 47.7% and W2: 399/789, 50.8%) who selected the difficulty of incorporating AI into clinical practice as the main barrier expressed their concern that the use of AI might be hampered by its misalignment with the specific context of clinical practice. This issue is associated with the generalizability and reproducibility of AI algorithms [20], which are often trained on clean datasets with no poor-quality information but are applied in a real clinical setting where data may be incomplete or erroneous. When comparing responses in both waves, this topic was more relevant for researchers in W2 than those in W1, which may indicate a growing concern about this misalignment. Furthermore, data accuracy issues may influence the quality of AI algorithm outputs by introducing biases [20,71,77,78] due to discrepancies in patient population (eg, individuals from low socioeconomic backgrounds and underrepresented minority groups) and organizational settings (eg, hospitals and primary care) [79,80].

Cost reduction is another potential benefit of AI in diagnostic medicine, as it relates to the optimization of resource use [72], processes, and services [71], as well as financial management [18,70]. As a result, the same therapy could be offered at a lower cost, or more services could be provided at the same cost. The use of AI to process large image datasets, for example, could offer workflow and productivity gains and reduce the workforce in many back-office activities, such as billing, clinical appointments, and staffing [72]. AI could have a variable impact on patient-, organizational-, and system-level tasks, potentially leading insurers to revise their reimbursement policy to reduce health care costs and improve quality [18]. Moreover, AI may make costly and time-consuming screening programs more affordable in resource-limited countries [23,81].

A total of 3 questions assessed the respondents’ beliefs about the changes in costs driven by AI. Most respondents (W1: 982/1360, 69.3% and W2: 1514/2243, 67.5%) in both waves anticipated that screening costs and physician workloads would be reduced in <10 years and patients’ hospitalization time would be shortened. These changes could potentially lower the cost of health care services and improve their quality, as physicians could perform more tasks concurrently and focus on more skilled work by delegating repetitive tasks to AI algorithms [18,72]. These benefits could be offset by the increased costs and risks for patients and the health system due to unnecessary testing and treatments prompted by AI [20]. Furthermore, there are concerns about the initial investments required for state-of-the-art AI products, the potential for long-term cost savings, and the need for a balanced evaluation of costs and benefits when considering AI integration [82,83].

Conversely, when appropriately implemented, AI can enhance diagnostic accuracy and reduce the need for redundant or follow-up tests. This efficiency is already leading to cost savings and a reduced burden on the health care system, and there is potential for further reduction of redundant and follow-up tests [84]. Therefore, AI value analyses should consider the technical aspects, short-term returns, and the overall value of automating processes [20].

The survey results in both waves have highlighted the anticipated transformation of AI in diagnostic instruments within a decade. In particular, image analysis and interpretation applications, such as x-ray, skin malignancy, and histopathologic diagnosis, have received optimistic perspectives. This may relate to advances in deep learning techniques for image analysis, such as convolutional neural networks and transformer architectures [85], which have significantly improved tasks such as image classification and object detection [3,29,46]. Deep learning is already the most used AI technique in health-related applications [71], and combining computing power, large datasets, and convolutional neural networks is also responsible for the subsequent revolution in medical imaging [18]. Since deep learning was introduced in 2012 for image recognition, it surpassed the human accuracy rate in specific, large data-labeled datasets after 5 years [72].

Furthermore, technology’s maturity may relate to perceptions of the use of AI in medical imaging. Digital medical images have been in use since the 1960s, and mature technologies have been built around them. Most radiology departments maintain picture archiving and communication systems containing historical images [18]. Cardiology applications highlighted by the respondents in both waves (eg, heart rhythm interpretation and disease diagnostics) have been in use for many decades, but recent developments are seen as paradigm shifts in clinical practice [86,87]. Because AI algorithms rely on the design of distinctive features to learn from data, more mature technologies are expected to provide a better set of such features, as is the case for these diagnostic instruments [79,88].

Conversely, the least optimistic results in both waves were related to applications that use other data formats, such as electronic health record texts (eg, identification of sepsis symptoms and prediction of clinical outcomes) or 1D signal data (eg, intrapartum monitoring and remote monitoring of gait). Multiple reasons may account for that, for example, data heterogeneity in electronic health records, including mixed data types, such as free text clinical notes, radiological reports, medication dosages, and medical codes [70,89]. Moreover, privacy policies and data interoperability impact information transfer across institutions that construct large datasets for deep learning representations of electronic health records [89]. For gait and intrapartum monitoring, some issues, such as the lack of large datasets and the use of clinical context as a complement for decision-making, also affect its performance [90].

Another factor that may help explain these results is regulation. In 2018, the Food and Drug Administration published a fast-track approval plan for AI medical algorithms for different applications [72]. Since then, the number of algorithms approved has soared, and there are now almost 400 algorithms associated with radiology and 58 associated with cardiology, many of which are dedicated to x-ray diagnostics and heart rhythm interpretation, respectively [10]. Thus, technological innovation and maturity combined with adequate regulation seem correlated to the respondents’ perceptions of AI’s impact on diagnostics in the coming years.

According to the respondents’ answers in both waves, the difficulty of incorporating AI into clinical practice, despite the reported benefits in terms of health outcomes, may hamper these innovations. These issues may be related to resistance to change in adopting AI in diagnostic medicine [91-93]. Another relevant challenge to AI adoption was ethical and regulatory issues. Accountability and legal responsibility for AI clinical decisions were marked as the most relevant issues in this topic in both waves. Accountability issues arise when AI algorithms make autonomous decisions about diagnoses and treatments beyond their role as support tools [20,23]. As respondents in W2 (941/2164, 43.5%) rated ethical and regulatory issues as more relevant than those in W1 (487/1296, 37.6%; in fact, as the most relevant), this seems to be a growing concern. As more AI applications become available to the public and cause social changes shortly, it is unsurprising that this issue increased in relevance from one wave to another. Large language models, such as ChatGPT, are examples of how AI has become more accessible to people and how this may raise more concerns over time [94,95]. For example, the United Kingdom’s Medicines and Healthcare Products Regulatory Agency points out some issues with incorporating large language models in medical devices. While it anticipates their use in health care soon, it emphasizes the need to uphold safety, effectiveness, and ethical standards [96].

In short, there are still doubts about who should be held responsible if a patient experiences an adverse event due to AI-based technology [34]. Insurers and regulators will have to be able to distinguish algorithmic errors from those resulting from misuse by the clinician, the organization, or even the patient. This issue is exacerbated by the black box nature of AI systems [20]. The increased use of AI in medicine will probably lead to legal challenges regarding medical negligence attributed to complex decision support systems [18]. Radiologist associations globally have been evaluating the ethical integration of AI in medical imaging. The Canadian Association of Radiologists underscores the potential of AI in medical imaging, emphasizing the importance of addressing its ethical and legal challenges [97]. Furthermore, the Royal Australian and New Zealand College of Radiologists has formulated ethical guidelines and deployment standards in response to AI’s rapid growth in radiology [98]. Simultaneously, a Joint European and North American Multisociety Statement, representing several radiological entities, emphasizes AI’s potential to enhance radiology efficiency and introduce systemic errors, advocating for ethical AI use that prioritizes patient well-being and transparent practices [99].

According to the respondents in both waves, other relevant issues are accessing and sharing large amounts of patient data. The use of big data raises concerns that may hamper the potential of AI in diagnostic medicine. These relate to data protection and confidentiality and the need to access the large volumes of data needed to train AI algorithms. Regarding data protection and confidentiality, key concerns include the data’s origin, obtaining patient consent, authorization for reuse, data ownership and responsibility, who can access and reuse the data, and the conditions under which the data can be used [20]. For example, a recent report from the European Union Agency for Cybersecurity on the pivotal role of AI in medical imaging diagnosis shows the importance of cybersecurity and privacy controls in properly addressing these many concerns [100]. Other relevant issues raised in the literature are the risk of the inappropriate use of databases and imprecise consent terms [34]. Technology-related obstacles include limited deidentification techniques and the need to integrate and standardize large amounts of data [34].

A shortage of well-annotated datasets for training AI algorithms is a key obstacle to the large-scale introduction of these systems. Furthermore, the absence of clear, standardized regulations could lead to the illicit collection of data from unknown sources [23]. These concerns relate to the very nature of machine learning technologies, as they need large amounts of training materials to be taught. On the other hand, as clinical data are gathered from multiple and diverse sources, it becomes easier to trace them to patients, threatening their privacy [49]. As ubiquitous data collection becomes commonplace, consensus must be reached for a consent framework to guide health-related data sharing [18]. In addition, there is growing concern about the lack of clinician involvement in the development of AI applications. A recent systematic review showed that developers typically consulted clinicians late in the design process, and many applications lacked validation against clinical expertise or detailed algorithm descriptions [101].

A particularly sensitive problem is iatrogenic risks related to a lack of transparency in the algorithm training processes. Before an AI algorithm can be unleashed in clinical practice, it has to be debugged, audited, simulated, and validated, along with prospective scrutiny [72]. Respondents identified these problems as major issues in both waves, with higher percentages in W2 (170/789, 21.5%). Besides the need for certification of AI systems, professionals, and teams, the rapid evolution of machine learning–based models presents an enormous challenge to regulation as more data are collected and used in algorithm development. A critical problem is how these updates should be evaluated and audited [18]. Quality control instruments for AI algorithms are needed to prevent abuse by AI system developers, as clinical decision support systems could be programmed to favor certain drugs, tests, or devices without users being aware of this manipulation. Transparency will be difficult to achieve if companies make their algorithms purposefully opaque for proprietary or financial reasons [34].

Another issue, which is quite present in the reviewed literature but received relatively little attention from the respondents (W1: 32/489, 6.5% and W2: 60/940, 6.4%) who answered this question, relates to embedded bias present in AI algorithms due to a lack of inclusion of minority individuals in datasets [72]. Such biases, resulting from underrepresenting minority groups and those considered vulnerable in the datasets used to develop AI systems, could reinforce discriminatory practices based on race, sex, or other features [34]. Machine learning datasets must be large, but the often-used clinical trial research databases are derived mainly from majority populations. The resulting algorithms may be more likely to fail when applied to underserved and possibly underrepresented patient groups [49]. Algorithms trained on health care datasets, which inherently mirror biases in health care spending, have been observed to exacerbate racial disparities in access to care within the United States and can affect medical diagnosis [102]. This problem will require careful attention from regulatory agencies [103].

### Limitations

This study relies on previous studies that have explored future scenarios of science and technology by surveying researchers [39,54-58,60]. However, it also inherits its limitations. The first one is the lack of diversity among respondents, who were identified and selected based on their publications in scientific journals. Therefore, most respondents (W1: 1007/1251, 80.5% and W2: 1607/2095, 76.7%) are researchers and professors affiliated with universities and research organizations. While their insights offer valuable academic and technological perspectives on AI use in diagnostic medicine, the relatively lower representation of clinicians might not capture the complete range of insights from the frontline of clinical practice. Considering that our respondents were sourced from scientific publications, a domain where professors and researchers are more commonly the authors, this was somewhat expected given our methodology.

Another limitation is the potential optimism bias of respondents. As researchers involved with AI and diagnostic medicine, they may have more positive expectations about the future of this technology than other groups (eg, patients, managers, businesspeople, and politicians). Nevertheless, they are among the most qualified to inform about future possibilities of AI use in diagnostic medicine as they are helping to advance the scientific and technological knowledge in the field. The self-attribution of knowledge level by the respondents in the questionnaire is another limitation. We cannot verify the accuracy of the respondents’ self-assessments or assign knowledge levels to them. Therefore, the self-assigned knowledge level reflects how the respondents perceive their own knowledge in the area. However, this study only includes participants who are authors of peer-reviewed scientific articles related to AI and diagnostic medicine indexed in WoS, which minimizes the risk of incorporating opinions from people with no knowledge of the topic.

A potential limitation of our study is the varied interpretations of the term “artificial intelligence” among respondents. AI is a broad and multifaceted concept, encompassing everything from machine learning and neural networks to natural language processing and specialized rule-based systems [1,2]. The use of thesaurus terms to identify relevant respondents and survey questions may have introduced biases based on how individuals conceptualize AI. This variation in understanding could influence responses, particularly regarding the anticipated impact and integration of AI into diagnostic medicine. Hence, future studies may benefit from providing a clearer, more uniform definition of AI to respondents, ensuring a more consistent interpretation across the survey population.

We included radiology-specific terms in the search for respondents because this field is one of the most likely to be affected by the advances of AI in diagnostics [104-106]. We acknowledge that there is a potential selection bias in extending the search to radiology experts. However, it is important to highlight that the query also included general terms, such as diagnostics, medicine, and medical, which mitigates this potential bias by engaging a more diverse range of diagnostic experts.

Furthermore, there is the possibility of regional demographic bias. Collecting respondents’ emails from scientific publications may introduce a bias toward specific demographic groups, possibly leading to an underrepresentation of experts from certain regions. Our methodology for identifying experts does not allow us to have demographic information on nonrespondents. The lack of this demographic information prevents an extensive analysis of nonresponse bias and demographic balance. However, we have included a regional breakdown of the responses in Multimedia Appendix 3 to address this concern and provide additional information. While slight percentage variations exist in the responses from each region, the overarching response patterns remain consistent across them.

### Conclusions

We reported the results of a global, 2-wave, cross-sectional survey of >3000 researchers with knowledge in diagnostic medicine. Most respondents (W1: 965/1410, 68.4% and W2: 1555/2324, 66.9%) believed that AI would transform diagnostic medicine radically in 10 years. Furthermore, they expected that AI would reduce screening costs and increase diagnostic reliability in this period and that x-ray diagnosis and heart rhythm interpretation are the most likely diagnostic tools to be integrated with AI. According to the respondents, the main general barriers to using AI in diagnostic medicine were the difficulty of incorporating AI into clinical practice and ethical or regulatory issues. While there were little changes in expectations when comparing the 2 waves, ethical and regulatory issues were more relevant to respondents in the second survey (941/2164, 43.5%), which indicates that this issue is becoming more urgent.

Moreover, adapting AI to the specific context of clinical practice and uncertainties about legal responsibility and accountability for AI-supported clinical decisions were the main specific barriers in both surveys. While our results provide a broad overview of the future of AI use in diagnostic medicine, they also pave the way for more detailed, focused research into specific aspects, enriching our understanding of this evolving domain. This may include an in-depth analysis of clinicians’ trust in AI applications in diagnostic medicine; further analysis of the integration of AI in specific diagnostic tools, such as x-ray diagnosis, heart rhythm interpretation, and skin malignancy diagnosis; or a cost analysis of AI implementation.

The use of AI in diagnostic medicine is rapidly evolving and can potentially transform the quality and cost of health care for patients. As AI continues to advance, it is essential to address these challenges and ensure that AI-based technologies are developed and deployed responsibly and ethically. The success of AI in diagnostic medicine will ultimately depend on the willingness of stakeholders to collaborate, innovate, and work together to ensure that these technologies are used to improve patient outcomes and enhance the overall quality of health care.

## Appendix

Multimedia Appendix 1 Checklist for Reporting Results of Internet E-Surveys.

Multimedia Appendix 2 Survey references.

Multimedia Appendix 3 Summarized and visual representation of the process of recruiting respondents and data collection.

Multimedia Appendix 4 Data by knowledge level with Mann-Whitney U test and marginal homogeneity test results.

Multimedia Appendix 5 Respondents’ publication analysis.

## Abbreviations

AI: artificial intelligence
MeSH: Medical Subject Headings
SCI: Science Citation Index
W1: wave 1
W2: wave 2
WoS: Web of Science

## Data Availability

The datasets generated or analyzed during this study are not publicly available due to its size but are available from the corresponding author on reasonable request.
