# Supplementary material for: Future Use of AI in Diagnostic Medicine: 2-Wave Cross-Sectional Survey Study
Source: J Med Internet Res. 2025 Feb 27;27:e53892. doi: 10.2196/53892 (PMC11907171; doi:10.2196/53892)
Supplement: Multimedia Appendix 1 [file jmir_v27i1e53892_app1.docx]

**Multimedia Appendix 1 - CHERRIES checklist**

| ***Item Category*** | ***Checklist Item*** | ***Explanation*** |
| --- | --- | --- |
| **Design** | Describe survey design | The survey is a two-wave cross-sectional survey targeting researchers with expertise in AI and diagnostic medicine. The sample frame was authors of scientific publications on AI and diagnostic medicine indexed in the Web of Science Core Collection (WoS) between 2015 and 2020 for Wave 1 and between 2020 and 2022 for Wave 2. The sample is not a convenience sample; instead, respondents were identified from relevant scientific publications. |
| **IRB (Institutional Review Board) approval and informed consent process** | IRB approval | The study followed Brazilian Resolution No. 510/2016, which exempts public opinion research with unidentified participants from IRB registration and evaluation. |
|  | Informed consent | Respondents were informed that the survey was for research purposes, no personal or sensitive data would be collected, participation was voluntary, and consent was implied by survey completion. These details were provided in the invitation and survey introduction. The participants were told the length of time of the survey, which data were stored, who the investigator was, and the purpose of the study. |
|  | Data protection | No personal information was collected. Responses were anonymous, and data confidentiality was ensured by the survey platform (SurveyMonkey). |
| **Development and pre-testing** | Development and testing | The questionnaire was developed based on a literature review of AI applications in diagnostic medicine. A pilot study with 2,000 researchers was conducted in Wave 1 to test the survey’s functionality, consistency, and internal logic. Feedback from 91 pilot respondents was collected, and no changes were required, allowing the pilot data to be included in the final analysis. |
| **Recruitment process and description of the sample having access to the questionnaire** | Open survey versus closed survey | This was a closed survey, only open to a predefined sample of researchers identified via scientific publications indexed in WoS. |
|  | Contact mode | Initial contact with potential participants was made via email, using personalized invitations sent to authors of relevant AI and diagnostic medicine papers. |
|  | Advertising the survey | No external advertising or announcements were made; only personalized email invitations were sent to selected respondents from the WoS database. |
| **Survey administration** | Web/E-mail | The survey was conducted via the SurveyMonkey platform. Invitations were emailed to respondents, and data were automatically collected through the platform. |
|  | Context | The survey was not posted on a public website. It was distributed directly to researchers via email, reducing the possibility of pre-selection bias due to website content. |
|  | Mandatory/voluntary | The survey was voluntary, with no mandatory requirement for participation. |
|  | Incentives | No incentives, monetary or otherwise, were offered to participants. |
|  | Time/Date | Wave 1 data were collected in September 2020, and Wave 2 data were collected between January and February 2023. |
|  | Randomization of items or questionnaires | No randomization of questionnaire items was used. |
|  | Adaptive questioning | Certain items were conditionally displayed based on responses. For example, respondents reporting no knowledge of diagnostic medicine were excluded from the main questionnaire. |
|  | Number of Items | The questionnaire was divided into six parts. The number of items per page varied based on the section. |
|  | Number of screens (pages) | The questionnaire was distributed over ten pages. |
|  | Completeness check | The survey platform allowed consistency and completeness checks before submission, ensuring mandatory items were filled in where applicable. Non-response options like “not applicable” were available. |
|  | Review step | Respondents were able to go back and change their answers before submitting their responses. However, once the responses were submitted, there was no review button or option to modify the answers. |
| **Response rates** | Unique site visitor | Not applicable, as this was an email-distributed survey to a predefined group of respondents. |
|  | View rate (Ratio of unique survey visitors/unique site visitors) | It is not applicable, as there was no public website hosting the survey. |
|  | Participation rate (Ratio of unique visitors who agreed to participate/unique first survey page visitors) | For Wave 1, 7.7% of invited participants completed the survey (1622 out of 20,952). For Wave 2, 4.6% of invited participants completed the survey (2606 out of 56,480). |
|  | Completion rate (Ratio of users who finished the survey/users who agreed to participate) | For Wave 1, 85.7% of participants who started the survey completed it (1,208 out of 1,622). For Wave 2, 74.2% completed it (1,724 out of 2,606). |
| **Preventing multiple entries from the same individual** | Cookies used | Multiple entries from the same individual were prevented by only allowing one answer for each email address. |
|  | IP check | Multiple entries from the same individual were prevented by only allowing one answer for each email address. |
|  | Log file analysis | No log file analysis was conducted. |
|  | Registration | The survey was available for each unique email address, and only one response was allowed per email address to prevent multiple entries from the same individual. |
| **Analysis** | Handling of incomplete questionnaires | Only completed questionnaires were analyzed. Respondents who did not proceed beyond the initial knowledge assessment were excluded from the final analysis. |
|  | Questionnaires submitted with an atypical timestamp | No atypical timespan was considered. |
|  | Statistical correction | No statistical corrections were made for non-representative samples. However, non-parametric tests (marginal homogeneity and Mann-Whitney U) were used to analyze differences between waves and knowledge groups. |
