# Supplementary material for: Future Use of AI in Diagnostic Medicine: 2-Wave Cross-Sectional Survey Study
Source: J Med Internet Res. 2025 Feb 27;27:e53892. doi: 10.2196/53892 (PMC11907171; doi:10.2196/53892)
Supplement: Multimedia Appendix 2 [file jmir_v27i1e53892_app2.docx]

Multimedia Appendix 2 – Survey references

| **Topic** | **Alternatives** | **References** |
| --- | --- | --- |
| Broad use of AI in the future | Likely before or after ten years; unlikely | ^1-4^ |
| Possible AI applications in diagnostic medicine in the future | Reduce physicians’ workload | ^5,6^ |
|  | More reliable diagnostics | ^4,7-14^ |
|  | Reduce patients' hospitalization time | ^15,16^ |
|  | Reduce screening cost | ^17,18^ |
|  | Improve access to care and follow-up services | ^19,20^ |
|  | Improve compliance with treatments | ^21,22^ |
|  | Assist in predicting the spread of diseases in the patients | ^23,24^ |
| Possible AI intelligence in diagnostic medicine | Heart rhythm interpretation | ^25,26^ |
|  | Histopathology image interpretation | ^27,28^ |
|  | Cardiovascular diseases diagnosis | ^29,30^ |
|  | Skin malignancy diagnosis | ^31,32^ |
|  | Fundus photographs interpretation | ^33,34^ |
|  | X-ray diagnosis | ^35,36^ |
|  | MRI brain interpretation | ^37,38^ |
|  | Obstetrics - intrapartum monitoring | ^39,40^ |
|  | Neurology - remote monitoring of gait | ^41^ |
|  | Inferring health status through wearable devices | ^14,42^ |
|  | Prediction of clinical outcomes based on the electronic health record | ^1,43^ |
|  | Identification of sepsis symptoms | ^44^ |
| Factors hampering AI adoption in diagnostic medicine | The difficulty of incorporation into clinical practice | ^45-47^ |
|  | Ethical or regulatory issues | ^7,17,19,48^ |
|  | Lack of improvement in medical diagnostic results | ^19,49^ |
|  | Impact of the technology on the workforce | ^1,2,14^ |

References

1. Davenport T, Kalakota R. The potential for artificial intelligence in healthcare. *Future Health Journal*. 2019.

2. He J, Baxter SL, Xu J, Xu J, Zhou X, Zhang K. The practical implementation of artificial intelligence technologies in medicine. *Nat Med*. 2019;25(1):30-36.

3. Topol EJ. High-performance medicine: the convergence of human and artificial intelligence. *Nat Med*. 2019;25(1):44-56.

4. Jiang F, Jiang Y, Zhi H, et al. Artificial intelligence in healthcare: past, present and future. *Stroke Vasc Neurol*. 2017;2(4):230-243.

5. Alexander A, Jiang A, Ferreira C, Zurkiya D. An Intelligent Future for Medical Imaging: A Market Outlook on Artificial Intelligence for Medical Imaging. *J Am Coll Radiol*. 2020;17(1 Pt B):165-170. doi:10.1016/j.jacr.2019.07.019

6. Zhou L-Q, Wang J-Y, Yu S-Y, et al. Artificial intelligence in medical imaging of the liver. *World J Gastroenterol*. 2019;25(6):672-682. doi:10.3748/wjg.v25.i6.672

7. Becker A. Artificial intelligence in medicine: What is it doing for us today? *Health Policy and Technology*. 2019;8(2):198-205.

8. Biot J. How will clinical practice be impacted by artificial intelligence? *Eur J Dermatol*. 2019;29(S1):8-10.

9. Comaniciu D, Engel K, Georgescu B, Mansi T. Shaping the future through innovations: From medical imaging to precision medicine. *Med Image Anal*. 2016;33:19-26. doi:10.1016/j.media.2016.06.016

10. Hamet P, Tremblay J. Artificial intelligence in medicine. *Metab Clin Exp*. 2017;69S:S36-S40.

11. Miller DD, Brown EW. Artificial Intelligence in Medical Practice: The Question to the Answer? *Am J Med*. 2018;131(2):129-133.

12. Nakata N. Recent technical development of artificial intelligence for diagnostic medical imaging. *Jpn J Radiol*. 2019;37(2):103-108. doi:10.1007/s11604-018-0804-6

13. Recht M, Bryan RN. Artificial Intelligence: Threat or Boon to Radiologists? *J Am Coll Radiol*. 2017;14(11):1476-1480. doi:10.1016/j.jacr.2017.07.007

14. Yu K-H, Beam AL, Kohane IS. Artificial intelligence in healthcare. *Nat Biomed Eng*. 2018;2(10):719-731.

15. Hilton CB, Milinovich A, Felix C, et al. Personalized predictions of patient outcomes during and after hospitalization using artificial intelligence. *NPJ Digit Med*. 2020;3:51. doi:10.1038/s41746-020-0249-z

16. Lopez-Jimenez F, Attia Z, Arruda-Olson AM, et al. Artificial Intelligence in Cardiology: Present and Future. *Mayo Clin Proc*. 2020;95(5):1015-1039. doi:10.1016/j.mayocp.2020.01.038

17. Pesapane F, Volonté C, Codari M, Sardanelli F. Artificial intelligence as a medical device in radiology: ethical and regulatory issues in Europe and the United States. *Insights Imaging*. 2018;9(5):745-753. doi:10.1007/s13244-018-0645-y

18. Powell AC. Impact of the Artificial Nudge. *Acad Radiol*. 2020;27(1):143-146. doi:10.1016/j.acra.2019.09.010

19. Alami H, Lehoux P, Auclair Y, et al. Artificial Intelligence and Health Technology Assessment: Anticipating a New Level of Complexity. *J Med Internet Res*. 2020;22(7):e17707. doi:10.2196/17707

20. Ellahham S. Artificial Intelligence: The Future for Diabetes Care. *Am J Med*. 2020;133(8):895-900. doi:10.1016/j.amjmed.2020.03.033

21. Bain EE, Shafner L, Walling DP, et al. Use of a Novel Artificial Intelligence Platform on Mobile Devices to Assess Dosing Compliance in a Phase 2 Clinical Trial in Subjects With Schizophrenia. *JMIR Mhealth Uhealth*. 2017;5(2):e18. doi:10.2196/mhealth.7030

22. Fogel AL, Kvedar JC. Artificial intelligence powers digital medicine. *NPJ Digit Med*. 2018;1:5. doi:10.1038/s41746-017-0012-2

23. Secinaro S, Calandra D, Secinaro A, Muthurangu V, Biancone P. The role of artificial intelligence in healthcare: a structured literature review. *BMC Med Inform Decis Mak*. 2021;21(1):125. doi:10.1186/s12911-021-01488-9

24. Wong ZSY, Zhou J, Zhang Q. Artificial Intelligence for infectious disease Big Data Analytics. *Infect Dis Health*. 2019;24(1):44-48. doi:10.1016/j.idh.2018.10.002

25. Attia ZI, Noseworthy PA, Lopez-Jimenez F, et al. An artificial intelligence-enabled ECG algorithm for the identification of patients with atrial fibrillation during sinus rhythm: a retrospective analysis of outcome prediction. *Lancet*. 2019;394(10201):861-867. doi:10.1016/S0140-6736(19)31721-0

26. Mincholé A, Rodriguez B. Artificial intelligence for the electrocardiogram. *Nat Med*. 2019;25(1):22-23. doi:10.1038/s41591-018-0306-1

27. Holzinger A, Langs G, Denk H, Zatloukal K, Müller H. Causability and explainability of artificial intelligence in medicine. *Wiley Interdiscip Rev Data Min Knowl Discov*. 2019;9(4):e1312. doi:10.1002/widm.1312

28. Niazi MKK, Parwani AV, Gurcan MN. Digital pathology and artificial intelligence. *Lancet Oncol*. 2019;20(5):e253-e261. doi:10.1016/S1470-2045(19)30154-8

29. Kagiyama N, Shrestha S, Farjo PD, Sengupta PP. Artificial Intelligence: Practical Primer for Clinical Research in Cardiovascular Disease. *J Am Heart Assoc*. 2019;8(17):e012788. doi:10.1161/JAHA.119.012788

30. Krittanawong C, Zhang H, Wang Z, Aydar M, Kitai T. Artificial Intelligence in Precision Cardiovascular Medicine. *J Am Coll Cardiol*. 2017;69(21):2657-2664. doi:10.1016/j.jacc.2017.03.571

31. Hekler A, Utikal JS, Enk AH, et al. Superior skin cancer classification by the combination of human and artificial intelligence. *Eur J Cancer*. 2019;120:114-121. doi:10.1016/j.ejca.2019.07.019

32. Zakhem GA, Motosko CC, Ho RS. How Should Artificial Intelligence Screen for Skin Cancer and Deliver Diagnostic Predictions to Patients? *JAMA Dermatol*. 2018;154(12):1383-1384. doi:10.1001/jamadermatol.2018.2714

33. Kapoor R, Walters SP, Al-Aswad LA. The current state of artificial intelligence in ophthalmology. *Surv Ophthalmol*. 2019;64(2):233-240. doi:10.1016/j.survophthal.2018.09.002

34. Rajalakshmi R, Subashini R, Anjana RM, Mohan V. Automated diabetic retinopathy detection in smartphone-based fundus photography using artificial intelligence. *Eye (Lond)*. 2018;32(6):1138-1144. doi:10.1038/s41433-018-0064-9

35. Qin C, Yao D, Shi Y, Song Z. Computer-aided detection in chest radiography based on artificial intelligence: a survey. *Biomed Eng Online*. 2018;17(1):113. doi:10.1186/s12938-018-0544-y

36. Qin ZZ, Sander MS, Rai B, et al. Using artificial intelligence to read chest radiographs for tuberculosis detection: A multi-site evaluation of the diagnostic accuracy of three deep learning systems. *Sci Rep*. 2019;9(1):15000. doi:10.1038/s41598-019-51503-3

37. Lee E-J, Kim Y-H, Kim N, Kang D-W. Deep into the Brain: Artificial Intelligence in Stroke Imaging. *J Stroke*. 2017;19(3):277-285. doi:10.5853/jos.2017.02054

38. Martín Noguerol T, Paulano-Godino F, Martín-Valdivia MT, Menias CO, Luna A. Strengths, Weaknesses, Opportunities, and Threats Analysis of Artificial Intelligence and Machine Learning Applications in Radiology. *J Am Coll Radiol*. 2019;16(9 Pt B):1239-1247. doi:10.1016/j.jacr.2019.05.047

39. Balayla J, Shrem G. Use of artificial intelligence (AI) in the interpretation of intrapartum fetal heart rate (FHR) tracings: a systematic review and meta-analysis. *Arch Gynecol Obstet*. 2019;300(1):7-14. doi:10.1007/s00404-019-05151-7

40. Desai GS. Artificial Intelligence: The Future of Obstetrics and Gynecology. *J Obstet Gynaecol India*. 2018;68(4):326-327. doi:10.1007/s13224-018-1118-4

41. Belić M, Bobić V, Badža M, Šolaja N, Đurić-Jovičić M, Kostić VS. Artificial intelligence for assisting diagnostics and assessment of Parkinson's disease-A review. *Clin Neurol Neurosurg*. 2019;184:105442. doi:10.1016/j.clineuro.2019.105442

42. Tian S, Yang W, Le Grange JM, Wang P, Huang W, Ye Z. Smart healthcare: making medical care more intelligent. *Global Health Journal*. 2019;3(3):62-65. doi:10.1016/j.glohj.2019.07.001

43. Norgeot B, Glicksberg BS, Trupin L, et al. Assessment of a Deep Learning Model Based on Electronic Health Record Data to Forecast Clinical Outcomes in Patients With Rheumatoid Arthritis. *JAMA Netw Open*. 2019;2(3):e190606. doi:10.1001/jamanetworkopen.2019.0606

44. van Steenkiste T, Ruyssinck J, Baets L de, et al. Accurate prediction of blood culture outcome in the intensive care unit using long short-term memory neural networks. *Artif Intell Med*. 2019;97:38-43. doi:10.1016/j.artmed.2018.10.008

45. Bi WL, Hosny A, Schabath MB, et al. Artificial intelligence in cancer imaging: Clinical challenges and applications. *CA Cancer J Clin*. 2019;69(2):127-157. doi:10.3322/caac.21552

46. Kelly CJ, Karthikesalingam A, Suleyman M, Corrado G, King D. Key challenges for delivering clinical impact with artificial intelligence. *BMC Med*. 2019;17(1):195. doi:10.1186/s12916-019-1426-2

47. Stead WW. Clinical Implications and Challenges of Artificial Intelligence and Deep Learning. *JAMA*. 2018;320(11):1107-1108. doi:10.1001/jama.2018.11029

48. Safdar NM, Banja JD, Meltzer CC. Ethical considerations in artificial intelligence. *Eur J Radiol*. 2020;122:108768. doi:10.1016/j.ejrad.2019.108768

49. Blum A, Zins M. Radiology: Is its future bright? *Diagn Interv Imaging*. 2017;98(5):369-371. doi:10.1016/j.diii.2017.04.002
