# Supplementary material for: Future Use of AI in Diagnostic Medicine: 2-Wave Cross-Sectional Survey Study
Source: J Med Internet Res. 2025 Feb 27;27:e53892. doi: 10.2196/53892 (PMC11907171; doi:10.2196/53892)

Multimedia Appendix 3 - Summarized and visual representation of the process of recruiting respondents and data collection


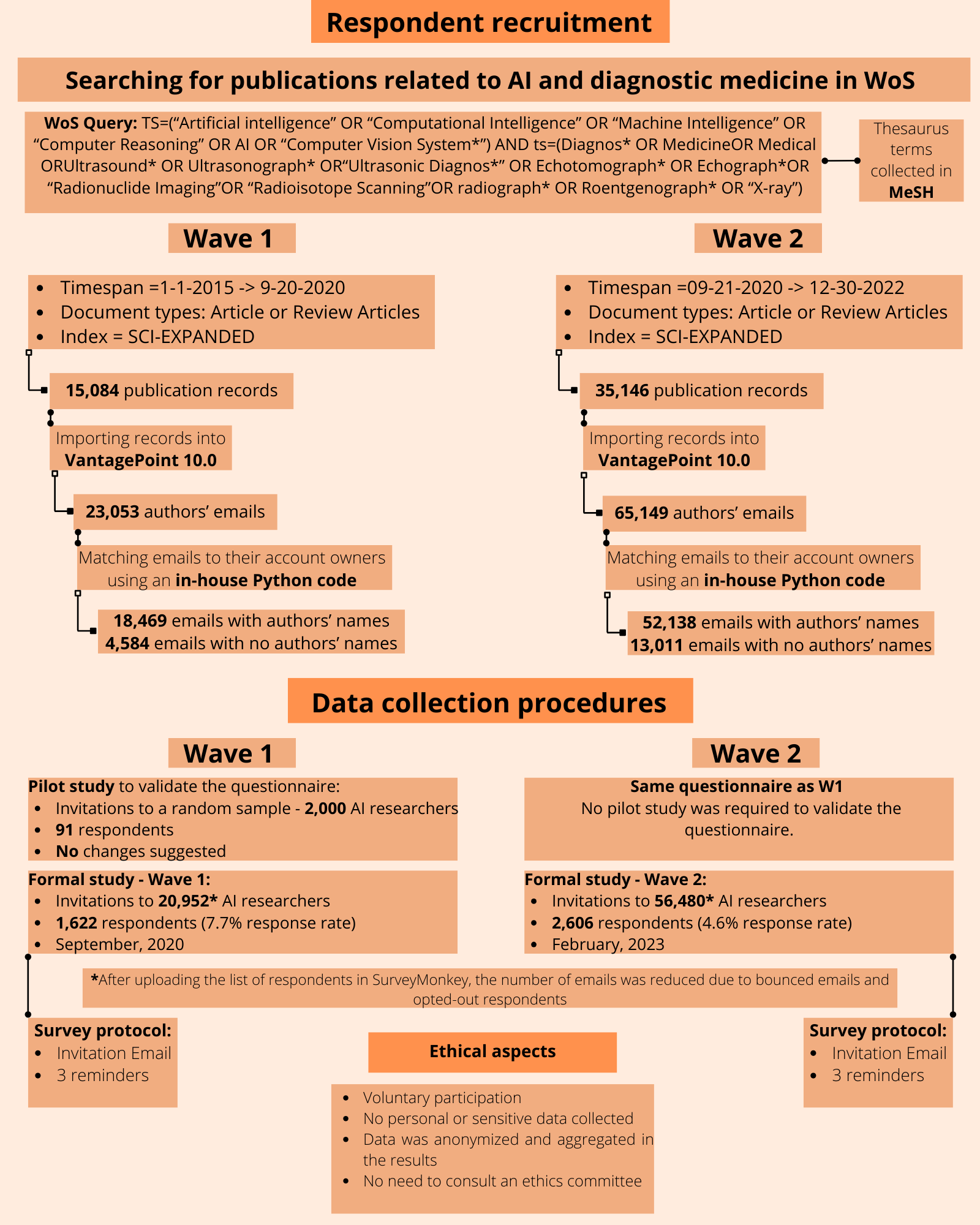

Supplement: Multimedia Appendix 3 [file jmir_v27i1e53892_app3.docx]
