# Supplementary material for: Future Use of AI in Diagnostic Medicine: 2-Wave Cross-Sectional Survey Study
Source: J Med Internet Res. 2025 Feb 27;27:e53892. doi: 10.2196/53892 (PMC11907171; doi:10.2196/53892)
Supplement: Multimedia Appendix 4 [file jmir_v27i1e53892_app4.docx]

Multimedia Appendix 4 - Data by knowledge level with Mann-Whitney U test and Marginal homogeneity test results

Note: P-value with * means that the variable is statistically significant at the 5% level.

| **Artificial intelligence will radically change diagnostic medicine** | | | | | | | | | **Likely after 10 years 2** | | **Likely before 10 years 1** | **Unlikely 3** | **Total** | |
| --- | --- | --- | --- | --- | --- | --- | --- | --- | --- | --- | --- | --- | --- | --- |
| **W1** | **I have good knowledge 1** | | | | | **Observations** | | | 197 | | 502 | 14 | 713 | |
|  |  |  |  |  |  | **%** | | | 27.63% | | 70.41% | 1.96% | 100.00% | |
|  | **I have some knowledge 2** | | | | | **Observations** | | | 221 | | 463 | 11 | 695 | |
|  |  |  |  |  |  | **%** | | | 31.80% | | 66.62% | 1.58% | 100.00% | |
| **W2** | **I have good knowledge 1** | | | | | **Observations** | | | 350 | | 798 | 32 | 1,180 | |
|  |  |  |  |  |  | **%** | | | 29.66% | | 67.63% | 2.71% | 100.00% | |
|  | **I have some knowledge 2** | | | | | **Observations** | | | 355 | | 756 | 31 | 1,142 | |
|  |  |  |  |  |  | **%** | | | 31.09% | | 66.20% | 2.71% | 100.00% | |
|  | | **Mann-Whitney U** | | | **P-value** | |  |  |  |  |  |  |  |  |
| **W1** | | 238,845.50 | | | 0.14743 | |  |  |  |  |  |  |  |  |
| **W2** | | 664,417.00 | | | 0.47954 | |  |  |  |  |  |  |  |  |
| **Marginal homogeneity test** | | | **Distinct Values** | **Observed MH Statistic** | | | | **Mean MH Statistic** | | **Statistic MH** | | **Standard statistic MH** | | **P-value** |
| **W1 & W2** | | | 3 | 986 | | | | 1006.5 | | 13.955 | | -1.469 | | 0.1418 |

| **Artificial intelligence will reduce physicians' workload** | | | | | | | | | **Likely after 10 years 2** | | **Likely before 10 years 1** | | **Unlikely 3** | **Total** | |
| --- | --- | --- | --- | --- | --- | --- | --- | --- | --- | --- | --- | --- | --- | --- | --- |
| **W1** | **I have good knowledge 1** | | | | **Observations** | | | | 158 | | 441 | | 91 | 690 | |
|  |  |  |  |  | **%** | | | | 22.90% | | 63.91% | | 13.19% | 100.00% | |
|  | **I have some knowledge 2** | | | | **Observations** | | | | 174 | | 432 | | 59 | 665 | |
|  |  |  |  |  | **%** | | | | 26.17% | | 64.96% | | 8.87% | 100.00% | |
| **W2** | **I have good knowledge 1** | | | | **Observations** | | | | 290 | | 701 | | 151 | 1,142 | |
|  |  |  |  |  | **%** | | | | 25.39% | | 61.38% | | 13.22% | 100.00% | |
|  | **I have some knowledge 2** | | | | **Observations** | | | | 289 | | 695 | | 120 | 1,104 | |
|  |  |  |  |  | **%** | | | | 26.18% | | 62.95% | | 10.87% | 100.00% | |
|  | | **Mann-Whitney U** | | | | **P-value** |  |  |  |  |  |  |  |  |  |
| **W1** | | 223,761.50 | | | | 0.35279 |  |  |  |  |  |  |  |  |  |
| **W2** | | 616,071.50 | | | | 0.27919 |  |  |  |  |  |  |  |  |  |
| **Marginal homogeneity test** | | | **Distinct Values** | **Observed MH Statistic** | | | | **Mean MH Statistic** | | **Statistic MH** | | **Standard statistic MH** | | | **P-value** |
| **W1 & W2** | | | 3 | 1176 | | | | 1201.5 | | 17.896 | | -1.425 | | | 0.1542 |

| **Artificial intelligence will provide more reliable diagnostics** | | | | | | | **Likely after 10 years 2** | | **Likely before 10 years 1** | | **Unlikely 3** | **Total** | |
| --- | --- | --- | --- | --- | --- | --- | --- | --- | --- | --- | --- | --- | --- |
| **W1** | **I have good knowledge 1** | | | **Observations** | | | 198 | | 474 | | 20 | 692 | |
|  |  |  |  | **%** | | | 28.61% | | 68.50% | | 2.89% | 100.00% | |
|  | **I have some knowledge 2** | | | **Observations** | | | 200 | | 452 | | 17 | 669 | |
|  |  |  |  | **%** | | | 29.90% | | 67.56% | | 2.54% | 100.00% | |
| **W2** | **I have good knowledge 1** | | | **Observations** | | | 331 | | 764 | | 42 | 1,137 | |
|  |  |  |  | **%** | | | 29.11% | | 67.19% | | 3.69% | 100.00% | |
|  | **I have some knowledge 2** | | | **Observations** | | | 359 | | 715 | | 31 | 1,105 | |
|  |  |  |  | **%** | | | 32.49% | | 64.71% | | 2.81% | 100.00% | |
|  | | **Mann-Whitney U** | | | **P-value** | |  |  |  |  |  |  |  |
| **W1** | | 229,630.00 | | | 0.75418 | |  |  |  |  |  |  |  |
| **W2** | | 614,968.50 | | | 0.29667 | |  |  |  |  |  |  |  |
| **Marginal homogeneity test** | | | **Distinct Values** | **Observed MH Statistic** | | **Mean MH Statistic** | | **Statistic MH** | | **Standard statistic MH** | | | **P-value** |
| **W1 & W2** | | | 3 | 958 | | 975 | | 13.964 | | -1.217 | | | 0.2235 |

| **Artificial intelligence will reduce patients' hospitalization time** | | | | | | | **Likely after 10 years 2** | | **Likely before 10 years 1** | | **Unlikely 3** | **Total** | |
| --- | --- | --- | --- | --- | --- | --- | --- | --- | --- | --- | --- | --- | --- |
| **W1** | **I have good knowledge 1** | | | **Observations** | | | 243 | | 344 | | 102 | 689 | |
|  |  |  |  | **%** | | | 35.27% | | 49.93% | | 14.80% | 100.00% | |
|  | **I have some knowledge 2** | | | **Observations** | | | 258 | | 326 | | 83 | 667 | |
|  |  |  |  | **%** | | | 38.68% | | 48.88% | | 12.44% | 100.00% | |
| **W2** | **I have good knowledge 1** | | | **Observations** | | | 390 | | 546 | | 197 | 1,133 | |
|  |  |  |  | **%** | | | 34.42% | | 48.19% | | 17.39% | 100.00% | |
|  | **I have some knowledge 2** | | | **Observations** | | | 406 | | 516 | | 177 | 1,099 | |
|  |  |  |  | **%** | | | 36.94% | | 46.95% | | 16.11% | 100.00% | |
|  | | **Mann-Whitney U** | | | **P-value** | |  |  |  |  |  |  |  |
| **W1** | | 229,125.00 | | | 0.92020 | |  |  |  |  |  |  |  |
| **W2** | | 620,346.50 | | | 0.87277 | |  |  |  |  |  |  |  |
| **Marginal homogeneity test** | | | **Distinct Values** | **Observed MH Statistic** | | **Mean MH Statistic** | | **Statistic MH** | | **Standard statistic MH** | | | **P-value** |
| **W1 & W2** | | | 3 | 1404 | | 1427.5 | | 18.688 | | -1.257 | | | 0.2086 |

| **Artificial intelligence will reduce screening cost** | | | | | | | **Likely after 10 years 2** | | **Likely before 10 years 1** | | **Unlikely 3** | **Total** | |
| --- | --- | --- | --- | --- | --- | --- | --- | --- | --- | --- | --- | --- | --- |
| **W1** | **I have good knowledge 1** | | | **Observations** | | | 152 | | 473 | | 65 | 690 | |
|  |  |  |  | **%** | | | 22.03% | | 68.55% | | 9.42% | 100.00% | |
|  | **I have some knowledge 2** | | | **Observations** | | | 147 | | 469 | | 53 | 669 | |
|  |  |  |  | **%** | | | 21.97% | | 70.10% | | 7.92% | 100.00% | |
| **W2** | **I have good knowledge 1** | | | **Observations** | | | 260 | | 773 | | 109 | 1,142 | |
|  |  |  |  | **%** | | | 22.77% | | 67.69% | | 9.54% | 100.00% | |
|  | **I have some knowledge 2** | | | **Observations** | | | 276 | | 741 | | 82 | 1,099 | |
|  |  |  |  | **%** | | | 25.11% | | 67.42% | | 7.46% | 100.00% | |
|  | | **Mann-Whitney U** | | | **P-value** | |  |  |  |  |  |  |  |
| **W1** | | 226,469.00 | | | 0.45909 | |  |  |  |  |  |  |  |
| **W2** | | 624,799.50 | | | 0.82854 | |  |  |  |  |  |  |  |
| **Marginal homogeneity test** | | | **Distinct Values** | **Observed MH Statistic** | | **Mean MH Statistic** | | **Statistic MH** | | **Standard statistic MH** | | | **P-value** |
| **W1 & W2** | | | 3 | 1093 | | 1107 | | 16.837 | | -0.831 | | | 0.4057 |

| **Artificial intelligence will improve access to care and follow-up services** | | | | | | | | | **Likely after 10 years 2** | | **Likely before 10 years 1** | | **Unlikely 3** | **Total** | |
| --- | --- | --- | --- | --- | --- | --- | --- | --- | --- | --- | --- | --- | --- | --- | --- |
| **W1** | **I have good knowledge 1** | | | | **Observations** | | | | 184 | | 429 | | 73 | 686 | |
|  |  |  |  |  | **%** | | | | 26.82% | | 62.54% | | 10.64% | 100.00% | |
|  | **I have some knowledge 2** | | | | **Observations** | | | | 202 | | 395 | | 67 | 664 | |
|  |  |  |  |  | **%** | | | | 30.42% | | 59.49% | | 10.09% | 100.00% | |
| **W2** | **I have good knowledge 1** | | | | **Observations** | | | | 321 | | 687 | | 133 | 1,141 | |
|  |  |  |  |  | **%** | | | | 28.13% | | 60.21% | | 11.66% | 100.00% | |
|  | **I have some knowledge 2** | | | | **Observations** | | | | 334 | | 635 | | 134 | 1,103 | |
|  |  |  |  |  | **%** | | | | 30.28% | | 57.57% | | 12.15% | 100.00% | |
|  | | **Mann-Whitney U** | | | | **P-value** | |  |  |  |  |  |  |  |  |
| **W1** | | 222,018.00 | | | | 0.35458 | |  |  |  |  |  |  |  |  |
| **W2** | | 613,352.50 | | | | 0.23708 | |  |  |  |  |  |  |  |  |
| **Marginal homogeneity test** | | | **Distinct Values** | **Observed MH Statistic** | | | **Mean MH Statistic** | | | **Statistic MH** | | **Standard statistic MH** | | | **P-value** |
| **W1 & W2** | | | 3 | 1209 | | | 1222 | | | 17.407 | | -0.747 | | | 0.4552 |

| **Artificial intelligence will improve compliance with treatments** | | | | | | | | | **Likely after 10 years 2** | | **Likely before 10 years 1** | | **Unlikely 3** | **Total** | |
| --- | --- | --- | --- | --- | --- | --- | --- | --- | --- | --- | --- | --- | --- | --- | --- |
| **W1** | **I have good knowledge 1** | | | | **Observations** | | | | 214 | | 366 | | 111 | 691 | |
|  |  |  |  |  | **%** | | | | 30.97% | | 52.97% | | 16.06% | 100.00% | |
|  | **I have some knowledge 2** | | | | **Observations** | | | | 245 | | 340 | | 80 | 665 | |
|  |  |  |  |  | **%** | | | | 36.84% | | 51.13% | | 12.03% | 100.00% | |
| **W2** | **I have good knowledge 1** | | | | **Observations** | | | | 349 | | 581 | | 209 | 1,139 | |
|  |  |  |  |  | **%** | | | | 30.64% | | 51.01% | | 18.35% | 100.00% | |
|  | **I have some knowledge 2** | | | | **Observations** | | | | 396 | | 551 | | 154 | 1,101 | |
|  |  |  |  |  | **%** | | | | 35.97% | | 50.05% | | 13.99% | 100.00% | |
|  | | **Mann-Whitney U** | | | | **P-value** | |  |  |  |  |  |  |  |  |
| **W1** | | 228,945.00 | | | | 0.90078 | |  |  |  |  |  |  |  |  |
| **W2** | | 618,556.50 | | | | 0.54382 | |  |  |  |  |  |  |  |  |
| **Marginal homogeneity test** | | | **Distinct Values** | **Observed MH Statistic** | | | **Mean MH Statistic** | | | **Statistic MH** | | **Standard statistic MH** | | | **P-value** |
| **W1 & W2** | | | 3 | 1385 | | | 1412 | | | 19.222 | | -1.405 | | | 0.1601 |

| **Artificial intelligence will assist in predicting the spread of diseases in the patients** | | | **Likely after 10 years 2** | **Likely before 10 years 1** | **Unlikely 3** | **Total** |
| --- | --- | --- | --- | --- | --- | --- |
| **W1** | **I have good knowledge 1** | **Observations** | 199 | 441 | 49 | 689 |
|  |  | **%** | 28.88% | 64.01% | 7.11% | 100.00% |
|  | **I have some knowledge 2** | **Observations** | 215 | 435 | 18 | 668 |
|  |  | **%** | 32.19% | 65.12% | 2.69% | 100.00% |
| **W2** | **I have good knowledge 1** | **Observations** | 365 | 703 | 75 | 1,143 |
|  |  | **%** | 31.93% | 61.50% | 6.56% | 100.00% |
|  | **I have some knowledge 2** | **Observations** | 359 | 694 | 51 | 1,104 |
|  |  | **%** | 32.52% | 62.86% | 4.62% | 100.00% |

|  | **Mann-Whitney U** | **P-value** |
| --- | --- | --- |
| **W1** | 224,086.00 | 0.31802 |
| **W2** | 618,216.00 | 0.33159 |

| **Marginal homogeneity test** | **Distinct Values** | **Observed MH Statistic** | **Mean MH Statistic** | **Statistic MH** | **Standard statistic MH** | **P-value** |
| --- | --- | --- | --- | --- | --- | --- |
| **W1 & W2** | 3 | 1026 | 1053.5 | 15.091 | -1.822 | 0.0684 |

| **Heart rhythm interpretation** | | | | | | | | | **Likely after 10 years 2** | | **Likely before 10 years 1** | | **Unlikely 3** | **Total** |
| --- | --- | --- | --- | --- | --- | --- | --- | --- | --- | --- | --- | --- | --- | --- |
| **W1** | **I have good knowledge 1** | | | | **Observations** | | | | 99 | | 562 | | 10 | 671 |
|  |  |  |  |  | **%** | | | | 14.75% | | 83.76% | | 1.49% | 100.00% |
|  | **I have some knowledge 2** | | | | **Observations** | | | | 129 | | 500 | | 12 | 641 |
|  |  |  |  |  | **%** | | | | 20.12% | | 78.00% | | 1.87% | 100.00% |
| **W2** | **I have good knowledge 1** | | | | **Observations** | | | | 165 | | 947 | | 10 | 1,122 |
|  |  |  |  |  | **%** | | | | 14.71% | | 84.40% | | 0.89% | 100.00% |
|  | **I have some knowledge 2** | | | | **Observations** | | | | 186 | | 851 | | 15 | 1,052 |
|  |  |  |  |  | **%** | | | | 17.68% | | 80.89% | | 1.43% | 100.00% |
|  | | **Mann-Whitney U** | | | | **P-value** | |  |  |  |  |  |  |  |
| **W1** | | 202,735.50 | | | | 0.00840* | |  |  |  |  |  |  |  |
| **W2** | | 569,153.50 | | | | 0.02844* | |  |  |  |  |  |  |  |
| **Marginal homogeneity test** | | | **Distinct Values** | **Observed MH Statistic** | | | **Mean MH Statistic** | | | **Statistic MH** | | **Standard statistic MH** | | **P-value** |
| **W1 & W2** | | | 3 | 570 | | | 547.5 | | | 10.356 | | 2.173 | | 0.02981* |

| **Histopathology image interpretation** | | | | | | | | | **Likely after 10 years 2** | | **Likely before 10 years 1** | | **Unlikely 3** | | **Total** |
| --- | --- | --- | --- | --- | --- | --- | --- | --- | --- | --- | --- | --- | --- | --- | --- |
| **W1** | **I have good knowledge 1** | | | | **Observations** | | | | 137 | | 523 | | 18 | | 678 |
|  |  |  |  |  | **%** | | | | 20.21% | | 77.14% | | 2.65% | | 100.00% |
|  | **I have some knowledge 2** | | | | **Observations** | | | | 169 | | 463 | | 9 | | 641 |
|  |  |  |  |  | **%** | | | | 26.37% | | 72.23% | | 1.40% | | 100.00% |
| **W2** | **I have good knowledge 1** | | | | **Observations** | | | | 280 | | 827 | | 16 | | 1,123 |
|  |  |  |  |  | **%** | | | | 24.93% | | 73.64% | | 1.42% | | 100.00% |
|  | **I have some knowledge 2** | | | | **Observations** | | | | 289 | | 750 | | 17 | | 1,056 |
|  |  |  |  |  | **%** | | | | 27.37% | | 71.02% | | 1.61% | | 100.00% |
|  | | **Mann-Whitney U** | | | | **P-value** | |  |  |  |  |  |  |  |  |
| **W1** | | 207,539.00 | | | | 0.06147 | |  |  |  |  |  |  |  |  |
| **W2** | | 577,345.00 | | | | 0.17116 | |  |  |  |  |  |  |  |  |
| **Marginal homogeneity test** | | | **Distinct Values** | **Observed MH Statistic** | | | **Mean MH Statistic** | | | **Statistic MH** | | **Standard statistic MH** | | **P-value** | |
| **W1 & W2** | | | 3 | 791 | | | 796.5 | | | 12.379 | | -0.444 | | 0.65684 | |

| **Cardiovascular diseases diagnosis** | | | | | | | | | **Likely after 10 years 2** | | **Likely before 10 years 1** | | **Unlikely 3** | **Total** | |
| --- | --- | --- | --- | --- | --- | --- | --- | --- | --- | --- | --- | --- | --- | --- | --- |
| **W1** | **I have good knowledge 1** | | | | **Observations** | | | | 190 | | 467 | | 17 | 674 | |
|  |  |  |  |  | **%** | | | | 28.19% | | 69.29% | | 2.52% | 100.00% | |
|  | **I have some knowledge 2** | | | | **Observations** | | | | 204 | | 422 | | 10 | 636 | |
|  |  |  |  |  | **%** | | | | 32.08% | | 66.35% | | 1.57% | 100.00% | |
| **W2** | **I have good knowledge 1** | | | | **Observations** | | | | 308 | | 773 | | 36 | 1,117 | |
|  |  |  |  |  | **%** | | | | 27.57% | | 69.20% | | 3.22% | 100.00% | |
|  | **I have some knowledge 2** | | | | **Observations** | | | | 299 | | 733 | | 19 | 1,051 | |
|  |  |  |  |  | **%** | | | | 28.45% | | 69.74% | | 1.81% | 100.00% | |
|  | | **Mann-Whitney U** | | | | **P-value** | |  |  |  |  |  |  |  |  |
| **W1** | | 208,824.00 | | | | 0.32191 | |  |  |  |  |  |  |  |  |
| **W2** | | 581,358.50 | | | | 0.63008 | |  |  |  |  |  |  |  |  |
| **Marginal homogeneity test** | | | **Distinct Values** | **Observed MH Statistic** | | | **Mean MH Statistic** | | | **Statistic MH** | | **Standard statistic MH** | | | **P-value** |
| **W1 & W2** | | | 3 | 836 | | | 835.5 | | | 12.718 | | 0.039 | | | 0.9686 |

| **Skin malignancy diagnosis** | | | | | | | | | **Likely after 10 years 2** | | **Likely before 10 years 1** | | **Unlikely 3** | **Total** | |
| --- | --- | --- | --- | --- | --- | --- | --- | --- | --- | --- | --- | --- | --- | --- | --- |
| **W1** | **I have good knowledge 1** | | | | **Observations** | | | | 111 | | 547 | | 18 | 676 | |
|  |  |  |  |  | **%** | | | | 16.42% | | 80.92% | | 2.66% | 100.00% | |
|  | **I have some knowledge 2** | | | | **Observations** | | | | 157 | | 477 | | 9 | 643 | |
|  |  |  |  |  | **%** | | | | 24.42% | | 74.18% | | 1.40% | 100.00% | |
| **W2** | **I have good knowledge 1** | | | | **Observations** | | | | 222 | | 880 | | 18 | 1,120 | |
|  |  |  |  |  | **%** | | | | 19.82% | | 78.57% | | 1.61% | 100.00% | |
|  | **I have some knowledge 2** | | | | **Observations** | | | | 237 | | 799 | | 18 | 1,054 | |
|  |  |  |  |  | **%** | | | | 22.49% | | 75.81% | | 1.71% | 100.00% | |
|  | | **Mann-Whitney U** | | | | **P-value** | |  |  |  |  |  |  |  |  |
| **W1** | | 203,613.00 | | | | 0.00611* | |  |  |  |  |  |  |  |  |
| **W2** | | 574,055.00 | | | | 0.12852 | |  |  |  |  |  |  |  |  |
| **Marginal homogeneity test** | | | **Distinct Values** | **Observed MH Statistic** | | | **Mean MH Statistic** | | | **Statistic MH** | | **Standard statistic MH** | | | **P-value** |
| **W1 & W2** | | | 3 | 702 | | | 701 | | | 11.895 | | 0.084 | | | 0.933 |

| **Fundus photographs interpretation** | | | | | | | | | **Likely after 10 years 2** | | **Likely before 10 years 1** | | **Unlikely 3** | **Total** | |
| --- | --- | --- | --- | --- | --- | --- | --- | --- | --- | --- | --- | --- | --- | --- | --- |
| **W1** | **I have good knowledge 1** | | | | **Observations** | | | | 128 | | 519 | | 13 | 660 | |
|  |  |  |  |  | **%** | | | | 19.39% | | 78.64% | | 1.97% | 100.00% | |
|  | **I have some knowledge 2** | | | | **Observations** | | | | 187 | | 421 | | 19 | 627 | |
|  |  |  |  |  | **%** | | | | 29.82% | | 67.15% | | 3.03% | 100.00% | |
| **W2** | **I have good knowledge 1** | | | | **Observations** | | | | 250 | | 836 | | 23 | 1,109 | |
|  |  |  |  |  | **%** | | | | 22.54% | | 75.38% | | 2.07% | 100.00% | |
|  | **I have some knowledge 2** | | | | **Observations** | | | | 267 | | 740 | | 26 | 1,033 | |
|  |  |  |  |  | **%** | | | | 25.85% | | 71.64% | | 2.52% | 100.00% | |
|  | | **Mann-Whitney U** | | | | **P-value** | |  |  |  |  |  |  |  |  |
| **W1** | | 183,133.00 | | | | 0.00000* | |  |  |  |  |  |  |  |  |
| **W2** | | 551,155.00 | | | | 0.04839* | |  |  |  |  |  |  |  |  |
| **Marginal homogeneity test** | | | **Distinct Values** | **Observed MH Statistic** | | | **Mean MH Statistic** | | | **Statistic MH** | | **Standard statistic MH** | | | **P-value** |
| **W1 & W2** | | | 3 | 787 | | | 778.5 | | | 12.379 | | 0.687 | | | 0.4923 |

| **X-ray diagnosis** | | | | | | | | | **Likely after 10 years 2** | | **Likely before 10 years 1** | | **Unlikely 3** | **Total** | |
| --- | --- | --- | --- | --- | --- | --- | --- | --- | --- | --- | --- | --- | --- | --- | --- |
| **W1** | **I have good knowledge 1** | | | | **Observations** | | | | 101 | | 564 | | 14 | 679 | |
|  |  |  |  |  | **%** | | | | 14.87% | | 83.06% | | 2.06% | 100.00% | |
|  | **I have some knowledge 2** | | | | **Observations** | | | | 123 | | 507 | | 12 | 642 | |
|  |  |  |  |  | **%** | | | | 19.16% | | 78.97% | | 1.87% | 100.00% | |
| **W2** | **I have good knowledge 1** | | | | **Observations** | | | | 192 | | 907 | | 24 | 1,123 | |
|  |  |  |  |  | **%** | | | | 17.10% | | 80.77% | | 2.14% | 100.00% | |
|  | **I have some knowledge 2** | | | | **Observations** | | | | 170 | | 873 | | 15 | 1,058 | |
|  |  |  |  |  | **%** | | | | 16.07% | | 82.51% | | 1.42% | 100.00% | |
|  | | **Mann-Whitney U** | | | | **P-value** | |  |  |  |  |  |  |  |  |
| **W1** | | 209,296.50 | | | | 0.06597 | |  |  |  |  |  |  |  |  |
| **W2** | | 583,080.50 | | | | 0.26613 | |  |  |  |  |  |  |  |  |
| **Marginal homogeneity test** | | | **Distinct Values** | **Observed MH Statistic** | | | **Mean MH Statistic** | | | **Statistic MH** | | **Standard statistic MH** | | | **P-value** |
| **W1 & W2** | | | 3 | 602 | | | 606 | | | 11.18 | | -0.358 | | | 0.7205 |

| **MRI brain interpretation** | | | | | | | | | **Likely after 10 years 2** | | **Likely before 10 years 1** | | **Unlikely 3** | **Total** | |
| --- | --- | --- | --- | --- | --- | --- | --- | --- | --- | --- | --- | --- | --- | --- | --- |
| **W1** | **I have good knowledge 1** | | | | **Observations** | | | | 186 | | 476 | | 17 | 679 | |
|  |  |  |  |  | **%** | | | | 27.39% | | 70.10% | | 2.50% | 100.00% | |
|  | **I have some knowledge 2** | | | | **Observations** | | | | 219 | | 402 | | 19 | 640 | |
|  |  |  |  |  | **%** | | | | 34.22% | | 62.81% | | 2.97% | 100.00% | |
| **W2** | **I have good knowledge 1** | | | | **Observations** | | | | 342 | | 746 | | 33 | 1,121 | |
|  |  |  |  |  | **%** | | | | 30.51% | | 66.55% | | 2.94% | 100.00% | |
|  | **I have some knowledge 2** | | | | **Observations** | | | | 346 | | 688 | | 21 | 1,055 | |
|  |  |  |  |  | **%** | | | | 32.80% | | 65.21% | | 1.99% | 100.00% | |
|  | | **Mann-Whitney U** | | | | **P-value** | |  |  |  |  |  |  |  |  |
| **W1** | | 201,533.50 | | | | 0.00561* | |  |  |  |  |  |  |  |  |
| **W2** | | 585,554.50 | | | | 0.63323 | |  |  |  |  |  |  |  |  |
| **Marginal homogeneity test** | | | **Distinct Values** | **Observed MH Statistic** | | | **Mean MH Statistic** | | | **Statistic MH** | | **Standard statistic MH** | | | **P-value** |
| **W1 & W2** | | | 3 | 959 | | | 965.5 | | | 13.775 | | -0.472 | | | 0.637 |

| **Obstetrics - intrapartum monitoring** | | | | | | | | | **Likely after 10 years 2** | | **Likely before 10 years 1** | | **Unlikely 3** | **Total** | |
| --- | --- | --- | --- | --- | --- | --- | --- | --- | --- | --- | --- | --- | --- | --- | --- |
| **W1** | **I have good knowledge 1** | | | | **Observations** | | | | 279 | | 325 | | 37 | 641 | |
|  |  |  |  |  | **%** | | | | 43.53% | | 50.70% | | 5.77% | 100.00% | |
|  | **I have some knowledge 2** | | | | **Observations** | | | | 286 | | 252 | | 56 | 594 | |
|  |  |  |  |  | **%** | | | | 48.15% | | 42.42% | | 9.43% | 100.00% | |
| **W2** | **I have good knowledge 1** | | | | **Observations** | | | | 436 | | 551 | | 96 | 1,083 | |
|  |  |  |  |  | **%** | | | | 40.26% | | 50.88% | | 8.86% | 100.00% | |
|  | **I have some knowledge 2** | | | | **Observations** | | | | 425 | | 494 | | 68 | 987 | |
|  |  |  |  |  | **%** | | | | 43.06% | | 50.05% | | 6.89% | 100.00% | |
|  | | **Mann-Whitney U** | | | | **P-value** | |  |  |  |  |  |  |  |  |
| **W1** | | 172,097.00 | | | | 0.00111* | |  |  |  |  |  |  |  |  |
| **W2** | | 533,302.00 | | | | 0.92397 | |  |  |  |  |  |  |  |  |
| **Marginal homogeneity test** | | | **Distinct Values** | **Observed MH Statistic** | | | **Mean MH Statistic** | | | **Statistic MH** | | **Standard statistic MH** | | | **P-value** |
| **W1 & W2** | | | 3 | 1086 | | | 1069 | | | 14.748 | | 1.153 | | | 0.249 |

| **Neurology - remote monitoring of gait** | | | | | | | | | **Likely after 10 years 2** | | **Likely before 10 years 1** | | **Unlikely 3** | **Total** | |
| --- | --- | --- | --- | --- | --- | --- | --- | --- | --- | --- | --- | --- | --- | --- | --- |
| **W1** | **I have good knowledge 1** | | | | **Observations** | | | | 250 | | 380 | | 25 | 655 | |
|  |  |  |  |  | **%** | | | | 38.17% | | 58.02% | | 3.82% | 100.00% | |
|  | **I have some knowledge 2** | | | | **Observations** | | | | 286 | | 302 | | 25 | 613 | |
|  |  |  |  |  | **%** | | | | 46.66% | | 49.27% | | 4.08% | 100.00% | |
| **W2** | **I have good knowledge 1** | | | | **Observations** | | | | 449 | | 599 | | 39 | 1,087 | |
|  |  |  |  |  | **%** | | | | 41.31% | | 55.11% | | 3.59% | 100.00% | |
|  | **I have some knowledge 2** | | | | **Observations** | | | | 426 | | 554 | | 35 | 1,015 | |
|  |  |  |  |  | **%** | | | | 41.97% | | 54.58% | | 3.45% | 100.00% | |
|  | | **Mann-Whitney U** | | | | **P-value** | |  |  |  |  |  |  |  |  |
| **W1** | | 183,642.50 | | | | 0.00274* | |  |  |  |  |  |  |  |  |
| **W2** | | 549,208.50 | | | | 0.84051 | |  |  |  |  |  |  |  |  |
| **Marginal homogeneity test** | | | **Distinct Values** | **Observed MH Statistic** | | | **Mean MH Statistic** | | | **Statistic MH** | | **Standard statistic MH** | | | **P-value** |
| **W1 & W2** | | | 3 | 976 | | | 970 | | | 13.91 | | 0.431 | | | 0.6662 |

| **Inferring health status through wearable devices** | | | | | | | | | **Likely after 10 years 2** | | **Likely before 10 years 1** | | **Unlikely 3** | **Total** | |
| --- | --- | --- | --- | --- | --- | --- | --- | --- | --- | --- | --- | --- | --- | --- | --- |
| **W1** | **I have good knowledge 1** | | | | **Observations** | | | | 177 | | 465 | | 18 | 660 | |
|  |  |  |  |  | **%** | | | | 26.82% | | 70.45% | | 2.73% | 100.00% | |
|  | **I have some knowledge 2** | | | | **Observations** | | | | 177 | | 433 | | 13 | 623 | |
|  |  |  |  |  | **%** | | | | 28.41% | | 69.50% | | 2.09% | 100.00% | |
| **W2** | **I have good knowledge 1** | | | | **Observations** | | | | 270 | | 796 | | 35 | 1,101 | |
|  |  |  |  |  | **%** | | | | 24.52% | | 72.30% | | 3.18% | 100.00% | |
|  | **I have some knowledge 2** | | | | **Observations** | | | | 267 | | 743 | | 23 | 1,033 | |
|  |  |  |  |  | **%** | | | | 25.85% | | 71.93% | | 2.23% | 100.00% | |
|  | | **Mann-Whitney U** | | | | **P-value** | |  |  |  |  |  |  |  |  |
| **W1** | | 204,075.00 | | | | 0.77459 | |  |  |  |  |  |  |  |  |
| **W2** | | 568,121.50 | | | | 0.96084 | |  |  |  |  |  |  |  |  |
| **Marginal homogeneity test** | | | **Distinct Values** | **Observed MH Statistic** | | | **Mean MH Statistic** | | | **Statistic MH** | | **Standard statistic MH** | | | **P-value** |
| **W1 & W2** | | | 3 | 797 | | | 791.5 | | | 12.679 | | 0.434 | | | 0.6644 |

| **Prediction of clinical outcomes based on the electronic health record** | | | | | | | | | **Likely after 10 years 2** | | **Likely before 10 years 1** | | **Unlikely 3** | **Total** | |
| --- | --- | --- | --- | --- | --- | --- | --- | --- | --- | --- | --- | --- | --- | --- | --- |
| **W1** | **I have good knowledge 1** | | | | **Observations** | | | | 200 | | 430 | | 30 | 660 | |
|  |  |  |  |  | **%** | | | | 30.30% | | 65.15% | | 4.55% | 100.00% | |
|  | **I have some knowledge 2** | | | | **Observations** | | | | 213 | | 398 | | 18 | 629 | |
|  |  |  |  |  | **%** | | | | 33.86% | | 63.28% | | 2.86% | 100.00% | |
| **W2** | **I have good knowledge 1** | | | | **Observations** | | | | 357 | | 705 | | 48 | 1,110 | |
|  |  |  |  |  | **%** | | | | 32.16% | | 63.51% | | 4.32% | 100.00% | |
|  | **I have some knowledge 2** | | | | **Observations** | | | | 320 | | 668 | | 44 | 1,032 | |
|  |  |  |  |  | **%** | | | | 31.01% | | 64.73% | | 4.26% | 100.00% | |
|  | | **Mann-Whitney U** | | | | **P-value** | |  |  |  |  |  |  |  |  |
| **W1** | | 205,070.00 | | | | 0.65513 | |  |  |  |  |  |  |  |  |
| **W2** | | 565,974.00 | | | | 0.57203 | |  |  |  |  |  |  |  |  |
| **Marginal homogeneity test** | | | **Distinct Values** | **Observed MH Statistic** | | | **Mean MH Statistic** | | | **Statistic MH** | | **Standard statistic MH** | | | **P-value** |
| **W1 & W2** | | | 3 | 961 | | | 974.5 | | | 14.396 | | -0.938 | | | 0.3484 |

| **Identification of sepsis symptoms** | | | | | | | | | **Likely after 10 years 2** | | **Likely before 10 years 1** | | **Unlikely 3** | **Total** | |
| --- | --- | --- | --- | --- | --- | --- | --- | --- | --- | --- | --- | --- | --- | --- | --- |
| **W1** | **I have good knowledge 1** | | | | **Observations** | | | | 191 | | 420 | | 40 | 651 | |
|  |  |  |  |  | **%** | | | | 29.34% | | 64.52% | | 6.14% | 100.00% | |
|  | **I have some knowledge 2** | | | | **Observations** | | | | 206 | | 350 | | 50 | 606 | |
|  |  |  |  |  | **%** | | | | 33.99% | | 57.76% | | 8.25% | 100.00% | |
| **W2** | **I have good knowledge 1** | | | | **Observations** | | | | 308 | | 707 | | 75 | 1,090 | |
|  |  |  |  |  | **%** | | | | 28.26% | | 64.86% | | 6.88% | 100.00% | |
|  | **I have some knowledge 2** | | | | **Observations** | | | | 358 | | 583 | | 63 | 1,004 | |
|  |  |  |  |  | **%** | | | | 35.66% | | 58.07% | | 6.27% | 100.00% | |
|  | | **Mann-Whitney U** | | | | **P-value** | |  |  |  |  |  |  |  |  |
| **W1** | | 183,263.00 | | | | 0.01135* | |  |  |  |  |  |  |  |  |
| **W2** | | 513,724.00 | | | | 0.00472* | |  |  |  |  |  |  |  |  |
| **Marginal homogeneity test** | | | **Distinct Values** | **Observed MH Statistic** | | | **Mean MH Statistic** | | | **Statistic MH** | | **Standard statistic MH** | | | **P-value** |
| **W1 & W2** | | | 3 | 999 | | | 988.5 | | | 14.705 | | 0.714 | | | 0.4752 |

| **Considering the following options, which one you believe is the most likely to hamper the use of artificial intelligence in diagnostic medicine?** | | | | | | **Ethical or regulatory issues 2** | | **Impact of the technology on the workforce 4** | | **Lack of improvement in medical diagnostic results 3** | | | **Other 5** | **The difficulty of incorporation into clinical practice 1** | | **Total** |
| --- | --- | --- | --- | --- | --- | --- | --- | --- | --- | --- | --- | --- | --- | --- | --- | --- |
| **W1** | **I have good knowledge 1** | | **Observations** | | | 250 | | 63 | | 48 | | | 47 | 258 | | 666 |
|  |  |  | **%** | | | 37.54% | | 9.46% | | 7.21% | | | 7.06% | 38.74% | | 100.00% |
|  | **I have some knowledge 2** | | **Observations** | | | 237 | | 48 | | 38 | | | 34 | 273 | | 630 |
|  |  |  | **%** | | | 37.62% | | 7.62% | | 6.03% | | | 5.40% | 43.33% | | 100.00% |
| **W2** | **I have good knowledge 1** | | **Observations** | | | 475 | | 80 | | 80 | | | 59 | 421 | | 1,115 |
|  |  |  | **%** | | | 42.60% | | 7.17% | | 7.17% | | | 5.29% | 37.76% | | 100.00% |
|  | **I have some knowledge 2** | | **Observations** | | | 465 | | 97 | | 76 | | | 43 | 367 | | 1,048 |
|  |  |  | **%** | | | 44.37% | | 9.26% | | 7.25% | | | 4.10% | 35.02% | | 100.00% |
|  | | **Mann-Whitney U** | | | **P-value** | |  |  |  |  |  |  |  |  |  |  |
| **W1** | | 196,249.00 | | | 0.03178* | |  |  |  |  |  |  |  |  |  |  |
| **W2** | | 568,699.50 | | | 0.25006 | |  |  |  |  |  |  |  |  |  |  |
| **Marginal homogeneity test** | | | | **Distinct Values** | | **Observed MH Statistic** | | | **Mean MH Statistic** | | **Statistic MH** | **Standard statistic MH** | | | **P-value** | |
| **W1 & W2** | | | | 5 | | 1797 | | | 1797.5 | | 28.138 | -0.018 | | | 0.9858 | |

| **Why do you believe artificial intelligence use in diagnostic medicine is likely to be hampered by the difficulty of incorporating it into clinical practice?** | | | **Aligning artificial intelligence to the specific context of clinical practice 3** | **Conflicts between artificial intelligence and other clinical strategies 1** | **Ignorance of the variables responsible for the artificial intelligence's decision 2** | **Other 4** | **Total** |
| --- | --- | --- | --- | --- | --- | --- | --- |
| **W1** | **I have good knowledge 1** | **Observations** | 125 | 51 | 57 | 22 | 255 |
|  |  | **%** | 49.02% | 20.00% | 22.35% | 8.63% | 100.00% |
|  | **I have some knowledge 2** | **Observations** | 127 | 66 | 66 | 14 | 273 |
|  |  | **%** | 46.52% | 24.18% | 24.18% | 5.13% | 100.00% |
| **W2** | **I have good knowledge 1** | **Observations** | 217 | 82 | 92 | 29 | 420 |
|  |  | **%** | 51.67% | 19.52% | 21.90% | 6.90% | 100.00% |
|  | **I have some knowledge 2** | **Observations** | 180 | 82 | 78 | 27 | 367 |
|  |  | **%** | 49.05% | 22.34% | 21.25% | 7.36% | 100.00% |

|  | **Mann-Whitney U** | **P-value** |
| --- | --- | --- |
| **W1** | 31,999.50 | 0.08525 |
| **W2** | 75,144.50 | 0.51207 |

| **Marginal homogeneity test** | **Distinct Values** | **Observed MH Statistic** | **Mean MH Statistic** | **Statistic MH** | **Standard statistic MH** | **P-value** |
| --- | --- | --- | --- | --- | --- | --- |
| **W1 & W2** | 4 | 294 | 307.5 | 8.675 | -1.556 | 0.1197 |

| **Why do you believe artificial intelligence use in diagnostic medicine is likely to be hampered by ethical or regulatory issues?** | | | | | **Algorithmic bias caused by the underrepresentation of minorities and underrated groups 4** | | **Difficulties to access, share and store large amounts of patient’s data 1** | **Difficulties to test, validate, certificate, and audit AI algorithms and systems 2** | **Other 6** | **Problems with financing, remuneration, reimbursement mechanisms and insurance models 5** | **Uncertainty about legal responsibility and accountability for AI-supported clinical decisions 3** | **Total** |
| --- | --- | --- | --- | --- | --- | --- | --- | --- | --- | --- | --- | --- |
| **W1** | **I have good knowledge 1** | | **Observations** | | 12 | | 57 | 39 | 3 | 15 | 123 | 249 |
|  |  |  | **%** | | 4.82% | | 22.89% | 15.66% | 1.20% | 6.02% | 49.40% | 100.00% |
|  | **I have some knowledge 2** | | **Observations** | | 19 | | 54 | 46 | 1 | 7 | 110 | 237 |
|  |  |  | **%** | | 8.02% | | 22.78% | 19.41% | 0.42% | 2.95% | 46.41% | 100.00% |
| **W2** | **I have good knowledge 1** | | **Observations** | | 27 | | 83 | 90 | 9 | 34 | 230 | 473 |
|  |  |  | **%** | | 5.71% | | 17.55% | 19.03% | 1.90% | 7.19% | 48.63% | 100.00% |
|  | **I have some knowledge 2** | | **Observations** | | 33 | | 95 | 97 | 8 | 12 | 219 | 464 |
|  |  |  | **%** | | 7.11% | | 20.47% | 20.91% | 1.72% | 2.59% | 47.20% | 100.00% |
|  | | **Mann-Whitney U** | | **P-value** | |  |  |  |  |  |  |  |
| **W1** | | 28,575.00 | | 0.51925 | |  |  |  |  |  |  |  |
| **W2** | | 102,287.00 | | 0.05450 | |  |  |  |  |  |  |  |

| **Marginal homogeneity test** | **Distinct Values** | **Observed MH Statistic** | **Mean MH Statistic** | **Statistic MH** | **Standard statistic MH** | **P-value** |
| --- | --- | --- | --- | --- | --- | --- |
| **W1 & W2** | 6 | 356 | 364 | 11.38 | -0.703 | 0.4821 |

| **Why do you believe artificial intelligence use in diagnostic medicine is likely to be hampered by a lack of improvement in medical diagnostic results?** | | | | | **Other 4** | **The intersection between multiple diagnoses 1** | | **Unrealistic expectations in patients regarding clinical outcomes 3** | | **Unsuitability to a real-world context of care and services 2** | **Total** | |
| --- | --- | --- | --- | --- | --- | --- | --- | --- | --- | --- | --- | --- |
| **W1** | **I have good knowledge 1** | | | **Observations** | 4 | 18 | | 14 | | 11 | 47 | |
|  |  |  |  | **%** | 8.51% | 38.30% | | 29.79% | | 23.40% | 100.00% | |
|  | **I have some knowledge 2** | | | **Observations** | 3 | 19 | | 5 | | 11 | 38 | |
|  |  |  |  | **%** | 7.89% | 50.00% | | 13.16% | | 28.95% | 100.00% | |
| **W2** | **I have good knowledge 1** | | | **Observations** | 7 | 27 | | 15 | | 31 | 80 | |
|  |  |  |  | **%** | 8.75% | 33.75% | | 18.75% | | 38.75% | 100.00% | |
|  | **I have some knowledge 2** | | | **Observations** | 9 | 32 | | 9 | | 26 | 76 | |
|  |  |  |  | **%** | 11.84% | 42.11% | | 11.84% | | 34.21% | 100.00% | |
|  | **Mann-Whitney U** | **P-value** | |  |  |  |  |  |  |  |  |  |
| **W1** | 744.50 | 0.16379 | |  |  |  |  |  |  |  |  |  |
| **W2** | 2,815.00 | 0.39841 | |  |  |  |  |  |  |  |  |  |
| **Marginal homogeneity test** | | | **Distinct Values** | | **Observed MH Statistic** | | **Mean MH Statistic** | | **Statistic MH** | **Standard statistic MH** | | **P-value** |
| **W1 & W2** | | | 4 | | 15 | | 15.5 | | 1.803 | -0.277 | | 0.7815 |

| **Why do you believe artificial intelligence use in diagnostic medicine is likely to be hampered by its impact on the workforce?** | | | **Difficulties in hiring, motivating and managing the trained professionals 2** | **Lack of educational resources (curriculum and continued education) available to train the professionals 3** | **Other 5** | **Problems with the certification of professionals 4** | **Reduction or obsolescence of jobs or decrease in compensation caused by new AI technologies 1** | **Total** |
| --- | --- | --- | --- | --- | --- | --- | --- | --- |
| **W1** | **I have good knowledge 1** | **Observations** | 11 | 19 | 2 | 3 | 26 | 61 |
|  |  | **%** | 18.03% | 31.15% | 3.28% | 4.92% | 42.62% | 100.00% |
|  | **I have some knowledge 2** | **Observations** | 13 | 10 | 2 | 5 | 18 | 48 |
|  |  | **%** | 27.08% | 20.83% | 4.17% | 10.42% | 37.50% | 100.00% |
| **W2** | **I have good knowledge 1** | **Observations** | 17 | 27 | 3 | 7 | 26 | 80 |
|  |  | **%** | 21.25% | 33.75% | 3.75% | 8.75% | 32.50% | 100.00% |
|  | **I have some knowledge 2** | **Observations** | 20 | 25 | 7 | 8 | 37 | 97 |
|  |  | **%** | 20.62% | 25.77% | 7.22% | 8.25% | 38.14% | 100.00% |

|  | **Mann-Whitney U** | **P-value** |
| --- | --- | --- |
| **W1** | 1,412.00 | 0.73855 |
| **W2** | 3,721.00 | 0.62506 |

| **Marginal homogeneity test** | **Distinct Values** | **Observed MH Statistic** | **Mean MH Statistic** | **Statistic MH** | **Standard statistic MH** | **P-value** |
| --- | --- | --- | --- | --- | --- | --- |
| **W1 & W2** | 5 | 2 | 3.5 | 1.118 | -1.342 | 0.1797 |

| **What is the highest degree of education you have completed?** | | | **Associate's degree 1** | **Bachelor's degree 2** | **Doctoral degree 4** | **Master’s degree 3** | **Total** |
| --- | --- | --- | --- | --- | --- | --- | --- |
| **W1** | **I have good knowledge 1** | **Observations** | 12 | 6 | 555 | 65 | 638 |
|  |  | **%** | 1.88% | 0.94% | 86.99% | 10.19% | 100.00% |
|  | **I have some knowledge 2** | **Observations** | 13 | 13 | 484 | 97 | 607 |
|  |  | **%** | 2.14% | 2.14% | 79.74% | 15.98% | 100.00% |
| **W2** | **I have good knowledge 1** | **Observations** | 17 | 15 | 915 | 124 | 1,071 |
|  |  | **%** | 1.59% | 1.40% | 85.43% | 11.58% | 100.00% |
|  | **I have some knowledge 2** | **Observations** | 13 | 33 | 802 | 168 | 1,016 |
|  |  | **%** | 1.28% | 3.25% | 78.94% | 16.54% | 100.00% |

| **Which occupation type best applies to you?** | | | **Manager, Executive 5** | **Masters, Ph.D. Student 1** | **Other 7** | **Physician, Clinician 3** | **Policymaker 6** | **Professor, Researcher 2** | **Public Health, Healthcare Professional 4** | **Total** |
| --- | --- | --- | --- | --- | --- | --- | --- | --- | --- | --- |
| **W1** | **I have good knowledge 1** | **Observations** | 14 | 45 | 17 | 113 | 0 | 441 | 10 | 640 |
|  |  | **%** | 2.19% | 7.03% | 2.66% | 17.66% | 0.00% | 68.91% | 1.56% | 100.00% |
|  | **I have some knowledge 2** | **Observations** | 9 | 92 | 23 | 20 | 1 | 460 | 4 | 609 |
|  |  | **%** | 1.48% | 15.11% | 3.78% | 3.28% | 0.16% | 75.53% | 0.66% | 100.00% |
| **W2** | **I have good knowledge 1** | **Observations** | 35 | 78 | 21 | 221 | 5 | 675 | 38 | 1,073 |
|  |  | **%** | 3.26% | 7.27% | 1.96% | 20.60% | 0.47% | 62.91% | 3.54% | 100.00% |
|  | **I have some knowledge 2** | **Observations** | 25 | 160 | 41 | 55 | 0 | 720 | 23 | 1,024 |
|  |  | **%** | 2.44% | 15.63% | 4.00% | 5.37% | 0.00% | 70.31% | 2.25% | 100.00% |

| **What type of institution do you work in?** | | | **Government 3** | **Hospital or similar organizations 2** | **Industry 4** | **University, Research Organization 1** | **Total** |
| --- | --- | --- | --- | --- | --- | --- | --- |
| **W1** | **I have good knowledge 1** | **Observations** | 11 | 105 | 35 | 491 | 642 |
|  |  | **%** | 1.71% | 16.36% | 5.45% | 76.48% | 100.00% |
|  | **I have some knowledge 2** | **Observations** | 16 | 43 | 34 | 516 | 609 |
|  |  | **%** | 2.63% | 7.06% | 5.58% | 84.73% | 100.00% |
| **W2** | **I have good knowledge 1** | **Observations** | 28 | 221 | 53 | 773 | 1,075 |
|  |  | **%** | 2.60% | 20.56% | 4.93% | 71.91% | 100.00% |
|  | **I have some knowledge 2** | **Observations** | 25 | 94 | 67 | 834 | 1,020 |
|  |  | **%** | 2.45% | 9.22% | 6.57% | 81.76% | 100.00% |

| **How many years of experience you have in your field?** | | | **Between 10 and 20 years 3** | **Between 5 and 10 years 2** | **Less than 5 years 2** | **More than 20 years 4** | **Total** |
| --- | --- | --- | --- | --- | --- | --- | --- |
| **W1** | **I have good knowledge 1** | **Observations** | 219 | 163 | 53 | 204 | 639 |
|  |  | **%** | 34.27% | 25.51% | 8.29% | 31.92% | 100.00% |
|  | **I have some knowledge 2** | **Observations** | 164 | 180 | 125 | 141 | 610 |
|  |  | **%** | 26.89% | 29.51% | 20.49% | 23.11% | 100.00% |
| **W2** | **I have good knowledge 1** | **Observations** | 363 | 246 | 81 | 383 | 1,073 |
|  |  | **%** | 33.83% | 22.93% | 7.55% | 35.69% | 100.00% |
|  | **I have some knowledge 2** | **Observations** | 313 | 264 | 166 | 281 | 1,024 |
|  |  | **%** | 30.57% | 25.78% | 16.21% | 27.44% | 100.00% |

| **In which region do you live?** | | | **Africa 3** | **Asia (including the Middle East) 1** | **Australasia or Pacific Islands 2** | **Europe 4** | **North America (including Central America and the Caribbean) 5** | **South America 6** | **Total** |
| --- | --- | --- | --- | --- | --- | --- | --- | --- | --- |
| **W1** | **I have good knowledge 1** | **Observations** | 14 | 177 | 12 | 262 | 132 | 43 | 640 |
|  |  | **%** | 2.19% | 27.66% | 1.88% | 40.94% | 20.63% | 6.72% | 100.00% |
|  | **I have some knowledge 2** | **Observations** | 20 | 190 | 20 | 196 | 126 | 59 | 611 |
|  |  | **%** | 3.27% | 31.10% | 3.27% | 32.08% | 20.62% | 9.66% | 100.00% |
| **W2** | **I have good knowledge 1** | **Observations** | 30 | 287 | 19 | 471 | 193 | 74 | 1,074 |
|  |  | **%** | 2.79% | 26.72% | 1.77% | 43.85% | 17.97% | 6.89% | 100.00% |
|  | **I have some knowledge 2** | **Observations** | 20 | 353 | 18 | 378 | 157 | 99 | 1,025 |
|  |  | **%** | 1.95% | 34.44% | 1.76% | 36.88% | 15.32% | 9.66% | 100.00% |
