# Supplementary material for: Future Use of AI in Diagnostic Medicine: 2-Wave Cross-Sectional Survey Study
Source: J Med Internet Res. 2025 Feb 27;27:e53892. doi: 10.2196/53892 (PMC11907171; doi:10.2196/53892)
Supplement: Multimedia Appendix 5 [file jmir_v27i1e53892_app5.docx]

Multimedia Appendix 5 - Respondents’ publication analysis
